# Metallothionein-1G suppresses pancreatic cancer cell stemness by limiting activin A secretion *via* NF-κB inhibition

**DOI:** 10.7150/thno.51976

**Published:** 2021-01-01

**Authors:** Kai Li, Zhicheng Zhang, Yu Mei, Qingzhu Yang, Shupei Qiao, Cheng Ni, Yao Yao, Xinyuan Li, Mengmeng Li, Dongdong Wei, Wangjun Fu, Xuefei Guo, Xuemei Huang, Huanjie Yang

**Affiliations:** 1 School of Life Science and Technology, Harbin Institute of Technology, Harbin, 150001, China.; 2 Department of General Surgery, Fourth Affiliated Hospital of Harbin Medical University, Harbin, 150001, China.

**Keywords:** MT1G, PDAC stemness, gemcitabine resistance, activin A, follistatin

## Abstract

Resistance to chemotherapy is a long-standing problem in the management of cancer, and cancer stem cells are regarded as the main source of this resistance. This study aimed to investigate metallothionein (MT)-1G involvement in the regulation of cancer stemness and provide a strategy to overcome chemoresistance in pancreatic ductal adenocarcinoma (PDAC).

**Methods:** MT1G was identified as a critical factor related with gemcitabine resistance in PDAC cells by mRNA microarray. Its effects on PDAC stemness were evaluated through sphere formation and tumorigenicity. LC-MS/MS analysis of conditional medium revealed that activin A, a NF-κB target, was a major protein secreted from gemcitabine resistant PDAC cells. Both loss-of-function and gain-of-function approaches were used to validate that MT1G inhibited NF-κB-activin A pathway. Orthotopic pancreatic tumor model was employed to explore the effects on gemcitabine resistance with recombinant follistatin to block activin A.

**Results:** Downregulation of *MT1G* due to hypermethylation of its promoter is related with pancreatic cancer stemness. Secretome analysis revealed that activin A, a NF-κB target, was highly secreted by drug resistant cells. It promotes pancreatic cancer stemness in Smad4-dependent or independent manners. Mechanistically, MT1G negatively regulates NF-κB signaling and promotes the degradation of NF-κB p65 subunit by enhancing the expression of E3 ligase TRAF7. Blockade of activin A signaling with follistatin could overcome gemcitabine resistance.

**Conclusions:** MT1G suppresses PDAC stemness by limiting activin A secretion *via* NF-κB inhibition. The blockade of the activin A signaling with follistatin may provide a promising therapeutic strategy for overcoming gemcitabine resistance in PDAC.

## Introduction

Pancreatic ductal adenocarcinoma (PDAC) is a devastating disease. Gemcitabine is the standard chemotherapeutic agent that has been widely used for the treatment of PDAC for over a decade. However, innate or acquired drug resistance leads to dismal survival rates which is the hallmark of this disease. Emerging evidence suggests that pancreatic carcinomas harbor a distinct subpopulation of putative cancer stem cells (CSCs) defined by their self-renewal, differentiation, exclusive *in vivo* tumorigenicity and driving metastasis abilities 1,2. Most importantly, CSCs are highly-resistant to conventional chemotherapy and radiotherapy, which renders them as a primary source for tumor recurrences post-treatment 2-4. Therefore, novel therapies capable of eliminating CSCs and overcoming gemcitabine resistance are urgently needed for PDAC treatment.

MT1G belongs to the MT superfamily, which are cysteine-rich proteins with high binding affinity to heavy metal ions 5,6. MT1G has been reported to suppress carcinogenesis 5, inhibit metastasis and promote differentiation 7,8, which are characteristics linked to CSCs. Unfortunately, MT1G involvement in the regulation of cancer stemness has not been fully validated and the underlying mechanism remains unclear. In this study, we identified MT1G as a critical molecule related with PDAC cancer stemness. Downregulation of MT1G conferred to enhanced cancer stemness properties in chemoresistant PDAC cells.

To understand how MT1G represses cancer stemness, we performed secretome analysis and found that nuclear factor kappa B (NF-κB) pathway was enriched. One downstream target of NF-κB, activin A was heavily secreted from chemoresistant PDAC cells with MT1G downregulation. Activin A has been reported as a key player in regulating cancer stemness and chemoresistance 9,10, hence we speculated that MT1G might suppress PDAC stemness by limiting activin A secretion. Given that MT family members are generally considered unable to regulate gene expression by directly binding to the promoter 11, we hypothesized that MT1G inhibits activin A secretion by affecting NF-κB. Our data demonstrate that MT1G promotes the degradation of NF-κB p65 subunit *via* tumor necrosis factor receptor-associated factor 7 (TRAF7), thus limiting activin A secretion and suppressing pancreatic cancer cell stemness.

## Results

### MT1G suppresses the pancreatic cancer stemness features

**Figure 1:**
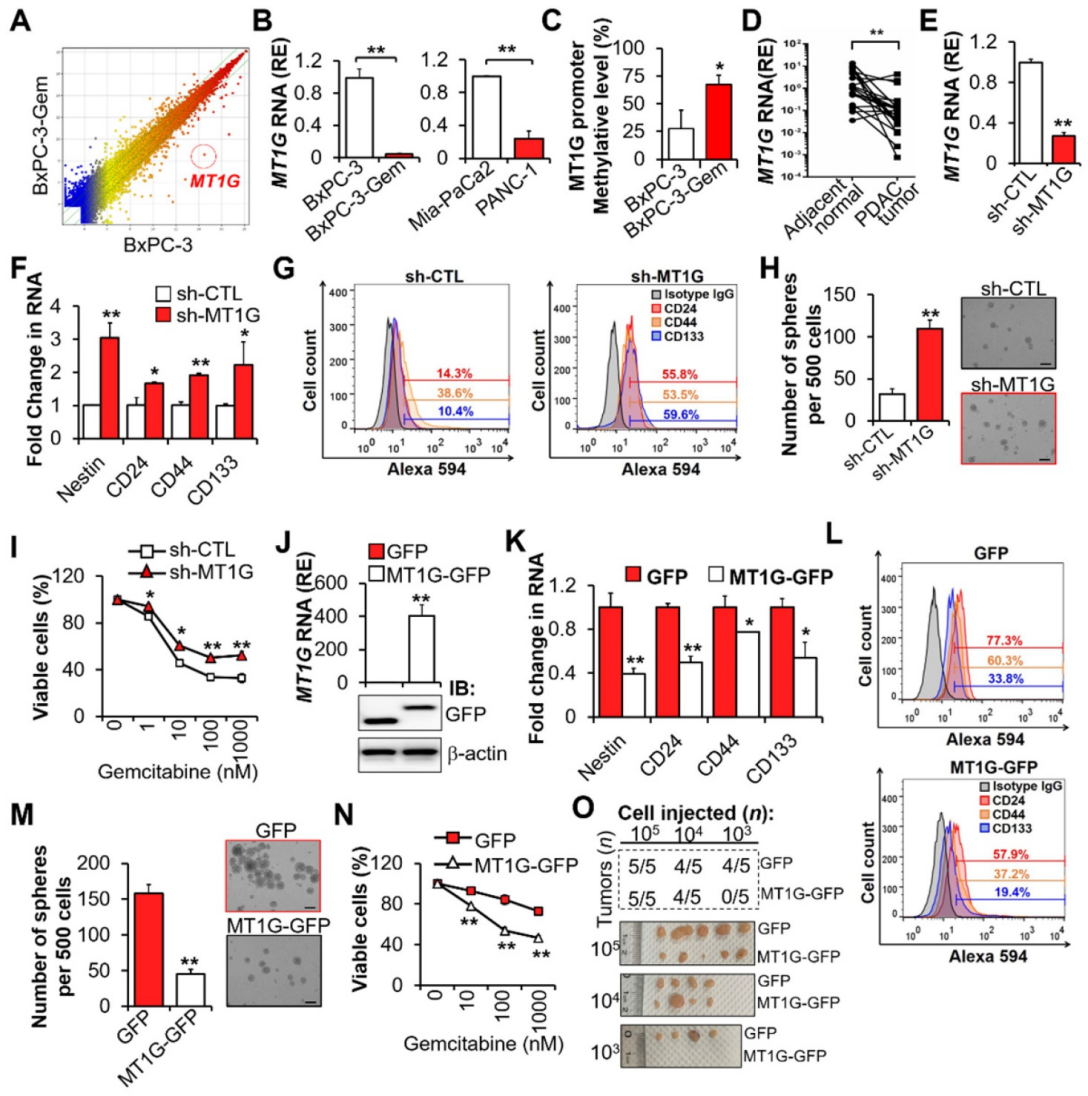
** Downregulation of *MT1G* in gemcitabine resistant PDAC cells confers to cancer stemness features.** (**A**) Scatterplot shows log intensities of global gene expression in BxPC-3 cells (x axis) against BxPC-3-Gem cells (y axis). The dot representing *MT1G* is shown. (**B**) RT-qPCR analysis of *MT1G* in indicated PDAC cells. (**C**) BSP analysis of the methylation status of *MT1G* promoter in BxPC-3 and BxPC-3-Gem cells. The percentage of methylation in each cell line is shown (n = 4). (**D**) RT-qPCR analysis of *MT1G* in PDAC and adjacent normal tissues (n = 21). (**E-I**) BxPC-3 cells were knocked down by shRNA, followed by RT-qPCR analysis (E, F), FACS analysis (G), sphere formation assay (H) and MTT assay post treatment with gemcitabine for 72 hours (I). Average number of spheres (H, left) and representative images (H, right) are shown. (**J-N**) RT-qPCR (J, up) and IB (J, bottom) analysis in MT1G overexpressing (MT1G-GFP) and control (GFP) BxPC-3-Gem cells. Expression of CSC markers (K, L), average number of spheres (M, left) and representative images (M, right), and relative cell viability after treatment with gemcitabine for 72 hours (N) were determined as described in E-I. (**O**) Summary table of *in vivo* tumor development in nude mice subcutaneously xenografted with a series of diluted MT1G overexpressing (MT1G-GFP) and control (GFP) BxPC-3-Gem cells (upper). Representative images of tumors formed (bottom) are shown. Data are presented as mean ± SD (n = 3). RE, relative expression. *P < 0.05, **P < 0.01 by two-tailed Student's *t* test.

To identify which factors are involved in the regulation of PDAC cell stemness and gemcitabine resistance, we performed mRNA array analysis 1,12, and found that *MT1G* was downregulated in chemoresistant BxPC-3-Gem (Figure S1A) compared with its parental BxPC-3 cells (Figure 1A; Table S1). Low expression levels of *MT1G* in gemcitabine resistant PDAC cells (Figure S1A) was further confirmed by RT-qPCR (Figure 1B). Bisulfite sequencing PCR (BSP) analysis revealed that *MT1G* promoter was hypermethylated in BxPC-3-Gem cells compared with the parental BxPC-3 cells (Figure 1C, Figure S1B). Moreover, *MT1G* was frequently downregulated in PDAC tumor tissues (17/21) compared with the adjacent counterparts (Figure 1D).

Previously, we found that the chemoresistant PDAC cells had CSCs properties 1. To clarify the function of MT1G in PDAC cell stemness, *MT1G* was stably knocked down in gemcitabine sensitive PDAC cells (Figure 1E, Figure S1C). Knockdown of *MT1G* led to increased expressions of cancer stemness markers (Figure 1F-G). Sphere formation assay showed that the sphero-forming ability, a trait of *in vitro* cancer stemness, was enhanced in *MT1G* knockdown cells (Figure 1H). PDAC cells' viability post gemcitabine treatment was also significantly increased after *MT1G* knockdown (Figure 1I, Figure S1D). Conversely, overexpression of *MT1G* in gemcitabine resistant PDAC cells (Figure 1J, Figure S1E) led to the loss of cancer stemness properties as shown by decreased cell markers expressions (Figure 1K-L), sphere formation ability (Figure 1M) and cells' viability in response to gemcitabine (Figure 1N, Figure S1F). Moreover, we observed an inverse correlation between stem cell markers and MT1G (Figure S1G). To examine pancreatic tumor initiation, limiting dilution assay (LDA) were conducted. Control or *MT1G* overexpressing BxPC-3-Gem cells were injected subcutaneously into immunocompromised mice at increasing dilutions and tumor incidence were recorded. No difference was found between two the groups at 10^5^ and 10^4^ cells injection. However, following injection with 10^3^ cells, 4/5 mice in the control group formed tumors compared with 0/5 mice in the *MT1G* overexpressing group (Figure 1O, Figure S1H). All the tumors were stained with CA19-9, a specific tumor marker (Figure S1I). *In vitro* LDA also showed that the sphere-initiating cell frequency was decreased upon overexpression of *MT1G* (Figure S1J). Taken together, these results indicate that the downregulation of *MT1G* in gemcitabine resistant pancreatic cancer cells is related with cancer stemness features.

### Low levels of ROS are required to maintain the hypermethylation of *MT1G* in pancreatic cancer stem cells

**Figure 2:**
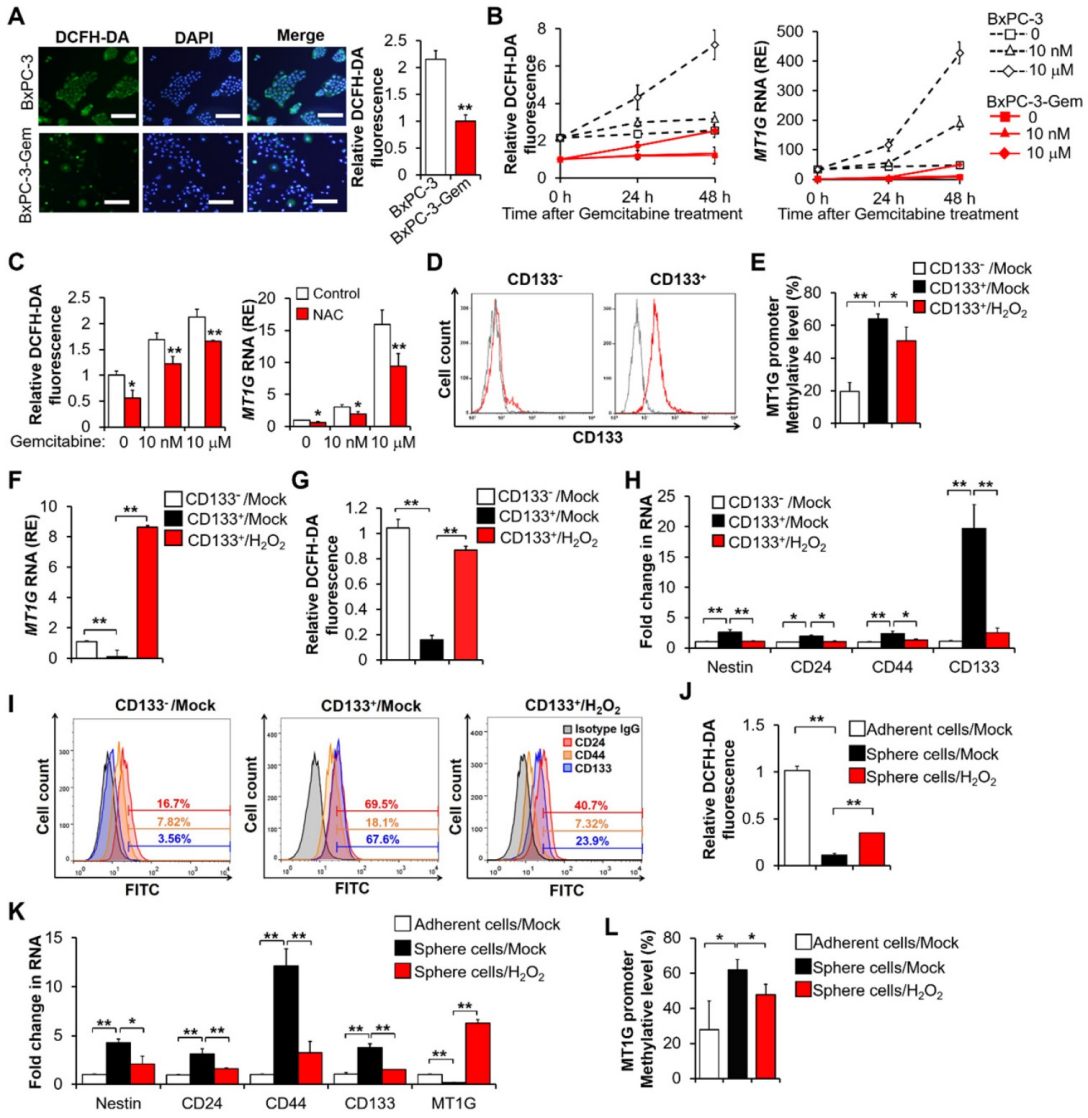
** Pancreatic CSCs exhibit hypermethylation of MT1G promoter.** (**A**) ROS levels were evaluated using DCFH-DA staining by fluorescence microscopy (left) and flow cytometry (right) in BxPC-3 and BxPC-3-Gem cells. Bar, 100 μm. (**B, C**) Relative ROS levels (B, C left) and MT1G expression (B, C right) were evaluated by flow cytometry using DCFH-DA and RT-qPCR in BxPC-3 and BxPC-3-Gem cells treated with indicated concentrations of gemcitabine for up to 48 hours (B) or BxPC-3 cells treated with NAC (5 mM) for 1 hour prior to gemcitabine treatment for 48 hours (C). (**D**) Representative flow-cytometry histograms of CD133 (red) and its respective isotype controls (gray) in separated CD133^-^ and CD133^+^ BxPC-3 cells subpopulation. (**E-I**) Separated CD133^+^ cells were treated with or without H_2_O_2_ (20 μM) for 72 hours, methylation status of *MT1G* promoter (E), mRNA expression of *MT1G* (F), relative ROS levels (G), and expression of CSC markers (H, I) were evaluated by BSP methylation analysis (E), RT-qPCR (F, H), flow cytometry using DCFH-DA (G) or indicated antibodies for CSC markers (I). (**J-L**) Four-weeks sphere cultured BxPC-3 cells were treated with or without H_2_O_2_ (20 μM) for 72 hours, and relative ROS levels (J), mRNA expression of CSC markers and *MT1G* (K), methylation status of *MT1G* promoter (L) were evaluated as described in E-I. Data are presented as mean ± SD (n = 3). RE, relative expression. *P < 0.05, **P < 0.01 by two-tailed Student's *t* test.

It has been reported that a low reactive oxygen species (ROS) level is required for the maintenance of stemness 13, thus we determined the intracellular ROS levels of gemcitabine resistant and sensitive PDAC cells by using a 2',7'-dichlorodihydrofluorescein diacetate (DCFH-DA) probe and found that the ROS levels were lower in the former (Figure 2A, Figure S2A). This result was consistent with the *MT1G* expression levels in the same cells (Figure 1B). Furthermore, we noticed that the generation of ROS increased with the induction of *MT1G* upon gemcitabine treatment (Figure 2B). Given the antioxidation function of MT1G, we should expect that the induction of MT1G would reduce ROS. However, these results suggest that the ROS generation triggers *MT1G* expression instead. To verify this hypothesis, we used N-acetyl-L-cysteine (NAC) to scavenge ROS and found that the expression of *MT1G* decreased along with reduction of the intracellular ROS levels (Figure 2C, Figure S2B), demonstrating that the expression of *MT1G* in PDAC cells is indeed induced by ROS generation.

As *MT1G* was hypermethylated in resistant cells (Figure 1C), we were interested in the possibility of ROS re-expressing *MT1G* of cancer stem cells through demethylation. The BxPC-3 cells were separated into CD133^+^ and CD133^-^ populations through magnetic-activated cell sorting (MACS) (Figure 2D). The CD133^+^ enriched cell population showed a higher level of *MT1G* methylation (Figure 2E, Figure S2C) and a lower level of *MT1G* mRNA expression (Figure 2F) compared to the CD133^-^ cell population. The CD133^+^ cells had low ROS level compared with the CD133^-^ cells (Figure 2G). After exposure to hydrogen peroxide (H_2_O_2_), the CD133^+^ cells exhibited increased ROS levels (Figure 2G) along with increased *MT1G* expression (Figure 2F) due to the reduction in hypermethylation of the *MT1G* promoter (Figure 2E, Figure S2C). Correspondingly, CD133^+^ cells lost CSCs property as shown by decreased expressions of CSCs markers after H_2_O_2_ exposure (Figure 2H-I). CD133^+^ population sorted from PANC-1 cells also showed low ROS level compared with the CD133^-^ cells, and increased ROS triggered MT1G upregulation as well as decreased expression of CSCs markers in those cells (Figure S2D-E). Enrichment of stem-cell subpopulations through sphere-forming culture showed similar results (Figure 2J-L). These results indicate that the low ROS levels can help preserve the hypermethylation of *MT1G* promoter to maintain the PDAC stemness features.

### MT1G negatively regulates activin A secretion

**Figure 3:**
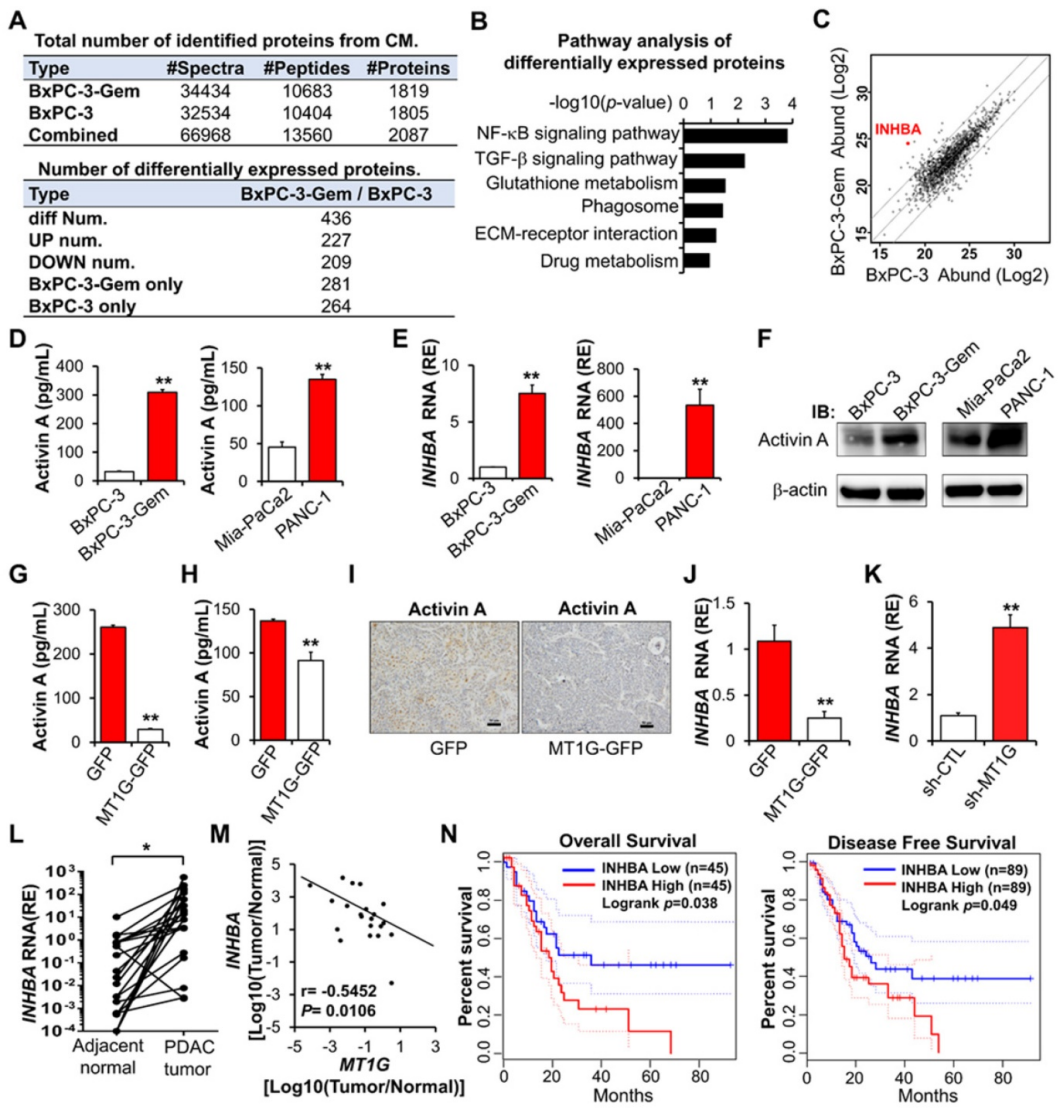
** Activin A is a downstream target of MT1G.** (**A**) Summary tables of secretome analysis results. (**B**) KEGG pathway analysis of differentially expressed proteins identified in BxPC-3 and BxPC-3-Gem CM. (**C**) Scatter plot of log intensities of secretome protein abundances between BxPC-3 cells (x axis) and BxPC-3-Gem cells (y axis). The dot representing INHBA is shown. (**D-F**) Secreted or cellular activin A protein levels and *INHBA* mRNA levels in pancreatic cancer cells were measured by ELISA (D), RT-qPCR (E) and IB (F). (**G, H**) ELISA analysis of secreted activin A protein levels in MT1G-overexpressing BxPC-3-Gem (G) and PANC-1 (H) cells. (**I**) Representative IHC images of activin A staining in the subcutaneous tumors from MT1G overexpressing (MT1G-GFP) and control (GFP) BxPC-3-Gem cells. Scale bar, 50 µm. (**J, K**) RT-qPCR analysis of *INHBA* mRNA levels in *MT1G* overexpressing BxPC-3-Gem cells (J) and *MT1G* knockdown BxPC-3 cells (K). (**L**) RT-qPCR analysis of *INHBA* mRNA expression in tumor and adjacent normal tissues of PDAC patients (n = 21). (**M**) Pearson's correlation analysis of *MT1G* and *INHBA* mRNA expression ratio (tumor/normal) from PDAC patients (n = 21). Pearson's correlation coefficient (r) and *p*-values are shown. (**N**) Kaplan-Meier curve of overall survival (left) and disease-free survival (right) of PDAC patients analyzed by using GEPIA. Blue curve represents patients with low expression of *INHBA*, red curve represents patients with high expression of *INHBA*. RE, relative expression. Data in (D, E, G, H, J, K) are presented as mean ± SD (n = 3), *P < 0.05, **P < 0.01 by two-tailed Student's *t* test.

To understand the underlying mechanism that MT1G suppresses PDAC stemness, we performed comparative secretome analysis using conditioned medium (CM) derived from BxPC-3-Gem cells and its parental BxPC-3 cells (Figure 3A, Table S2). Pathway analysis indicated that the differentially secreted peptides were enriched in both NF-κB and TGF-β signaling pathways (Figure 3B). INHBA (inhibin-β_A_, encoded by *INHBA* gene) was selected from these secreted proteins as it was on the top of the upregulated peptides in BxPC-3-Gem cells and is also a NF-κB regulated protein which is related with cancer stemness 9 (Figure 3C, Table S2). Indeed, we found that the mRNA level of *INHBA* was upregulated in cancer stem cells (spheres or CD133^+^ population cells) compared with the non-stem cancer cells (adherent or CD133^-^ cells) (Figure S3A). INHBA is a subunit of both activin and inhibin 14. Homodimer of INHBA forms activin A and heterodimer of INHBA and INHBB (inhibin-β_B_, encoded by *INHBB* gene) forms activin AB, while heterodimerization of INHBA with INHA (inhibin-α, encoded by *INHA* gene) results in inhibin A (Figure S3B)*.* Comparative secretome analysis revealed no significant difference in INHBB and INHA subunits, and further RT-qPCR analysis also indicated that there was no increased expression of *INHA* and *INHBB* in BxPC-3-Gem cells compared with BxPC-3 cells (Figure S3C). These results suggest that activin A, but not other inhibin and activin members, is heavily secreted from gemcitabine resistant PDAC cells.

Enzyme-linked immunosorbent assay (ELISA) confirmed that the secreted activin A levels were higher in gemcitabine resistant PDAC cells when compared with sensitive cells (Figure 3D), which was consistent to their intracellular mRNA and protein levels (Figure 3E-F). Then, we determined whether MT1G could affect the secretion of activin A and found that it negatively regulated the level of secreted activin A in PDAC cells (Figure 3G-H). IHC staining showed that MT1G decreased the protein level of activin A in subcutaneous tumors (Figure 3I). Furthermore, overexpression of *MT1G* suppressed *INHBA* expression while knockdown of *MT1G* promoted its expression (Figure 3J-K). Negative correlation between *MT1G* and *INHBA* was also observed in PDAC clinical samples, in which *MT1G* was down-regulated while *INHBA* was up-regulated in tumor tissues compared with the adjacent counterparts (Figure 3L-M, Figure 1D). Moreover, Kaplan-Meier survival analysis indicated that high expression of activin A in PDAC tissues was associated with a shorter overall survival, and disease-free survival of PDAC patients (Figure 3N).

### Activin A signaling contributes to PDAC stemness

**Figure 4:**
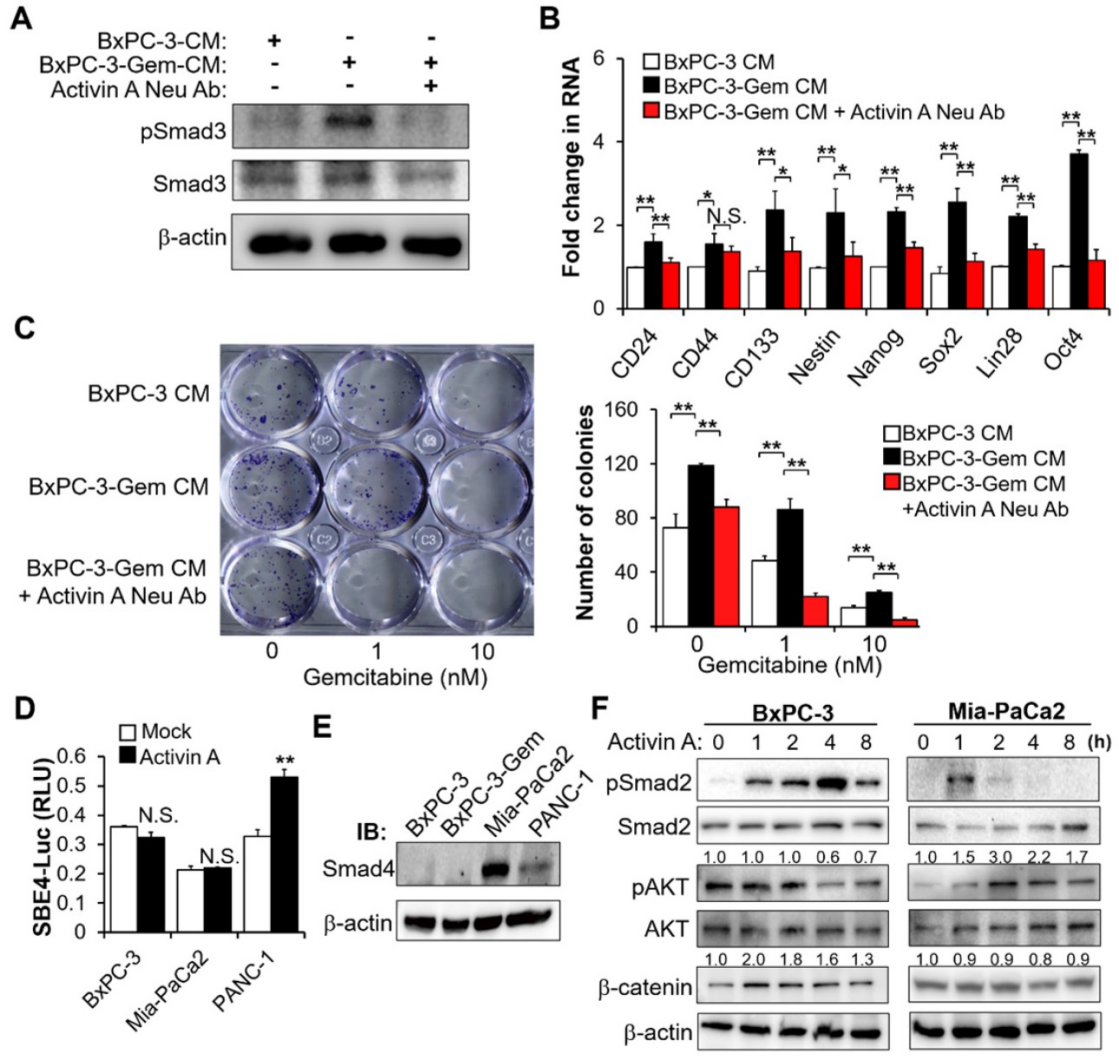
** Secreted activin A enhances PDAC stemness.** (**A-C**) BxPC-3 cells were treated with indicated CM for 1 hour (A) or 48 hours (B-C) in the presence or absence of neutralizing antibody of activin A (4 μg/mL), and then Smad activation was measured by IB with the indicated antibodies (A), mRNA expressions of indicated CSC markers were analyzed by RT-qPCR (B), cancer cell survival in response to gemcitabine was determined by colony formation assay (C). (**D**) Luciferase activity assay in PDAC cells transfected with SBE4-Luc reporter vector for 24 hours, followed by treatment with or without activin A (10 ng/mL) for 16 hours. (**E**) IB analysis of Smad4 expression in 4 PDAC cell lines. (**F**) IB analysis of the indicated proteins in BxPC-3 or Mia-PaCa2 cells treated with activin A (10 ng/mL) for up to 8 hours. Data are presented as mean ± SD (n = 3) and are representative of at least three independent experiments. *P < 0.05, **P < 0.01 by two-tailed Student's *t* test. N.S., not significant.

To examine whether the secreted activin A from gemcitabine resistant PDAC cells is a key mediator of PDAC stemness, we treated BxPC-3 cells with the CM from BxPC-3-Gem cells with and without activin A neutralizing antibody pretreatment, using BxPC-3 parental CM as control. Activin A signaling was triggered by CM from BxPC-3-Gem (but not parental cells), as shown by the increased phosphorylation of Smad3 (Figure 4A), a critical mediator of TGFβ signaling 15. This effect was blocked with pretreatment of activin A neutralizing antibody (Figure 4A). Correspondingly, the neutralizing antibody effectively reduced the expressions of CSCs markers which were induced by the treatment of CM from resistant cells (Figure 4B). Only *CD44* could not be restored by the antibody (Figure 4B). Furthermore, blocking of activin A signaling with the neutralizing antibody enhanced gemcitabine efficacy, showing that the neutralizing antibody could reduce cells' colony formation ability which was increased due to the resistant cell CM treatment (Figure 4C). These data indicate that activin A is an important secreted molecule which contributes to PDAC stemness. To further confirm the involvement of MT1G in regulating activin A secretion and its downstream signaling, we treated BxPC-3 cells with CM from *MT1G* knockdown or overexpression cells. Like as activin A treatment, cells treated with CM from *MT1G* knockdown BxPC-3 cells significantly increased phosphorylation of Smad3 (Figure S4A), expressions of CSCs markers (Figure S4B), as well as cells' colony formation ability (Figure S4C). Conversely, CM from *MT1G*-overexpressing BxPC-3-Gem cells exhibited inhibitory effect on activation of activin A signaling, expression of cancer stemness genes, and colony formation (Figure S4D-F).

In the canonical activin A signal transduction pathway, activin/TGF induces Smad2/3 phosphorylation and subsequent nuclear translocation with Smad4 to regulate target gene transcription 16. Whether activin A promotes the transcriptions of target cancer stemness genes through the canonical pathway was investigated. The Smad-binding element luciferase (SBE-Luc) reporter assays indicated that activin A could induce Smad signaling in PANC-1 cells (Figure 4D). Activin A induced canonical Smad pathway in PANC-1 cells was consistent with the Smad4 wild type status in this cell line (Figure 4E). However, it failed to induce Smad signaling in Mia-PaCa2 cells with Smad4 as well as BxPC-3 cells without Smad4 (Figure 4D-E). Wnt-catenin and AKT are two mediators of activin A signaling 17,18, thus we further analyzed Smad-independent pathway and found that activin A caused the induction of β-catenin and phosphorylation of AKT in BxPC-3 and Mia-PaCa2 cells, respectively (Figure 4F). PDAC is highly heterogeneous, with ~50% inactivation of Smad4 *via* mutation or deletion 19. These results indicate that activin A can extensively regulate cancer stemness in both Smad-dependent and independent manners in pancreatic cancer.

### MT1G inhibits activin A secretion by promoting ubiquitinated degradation of NF-κB p65

**Figure 5:**
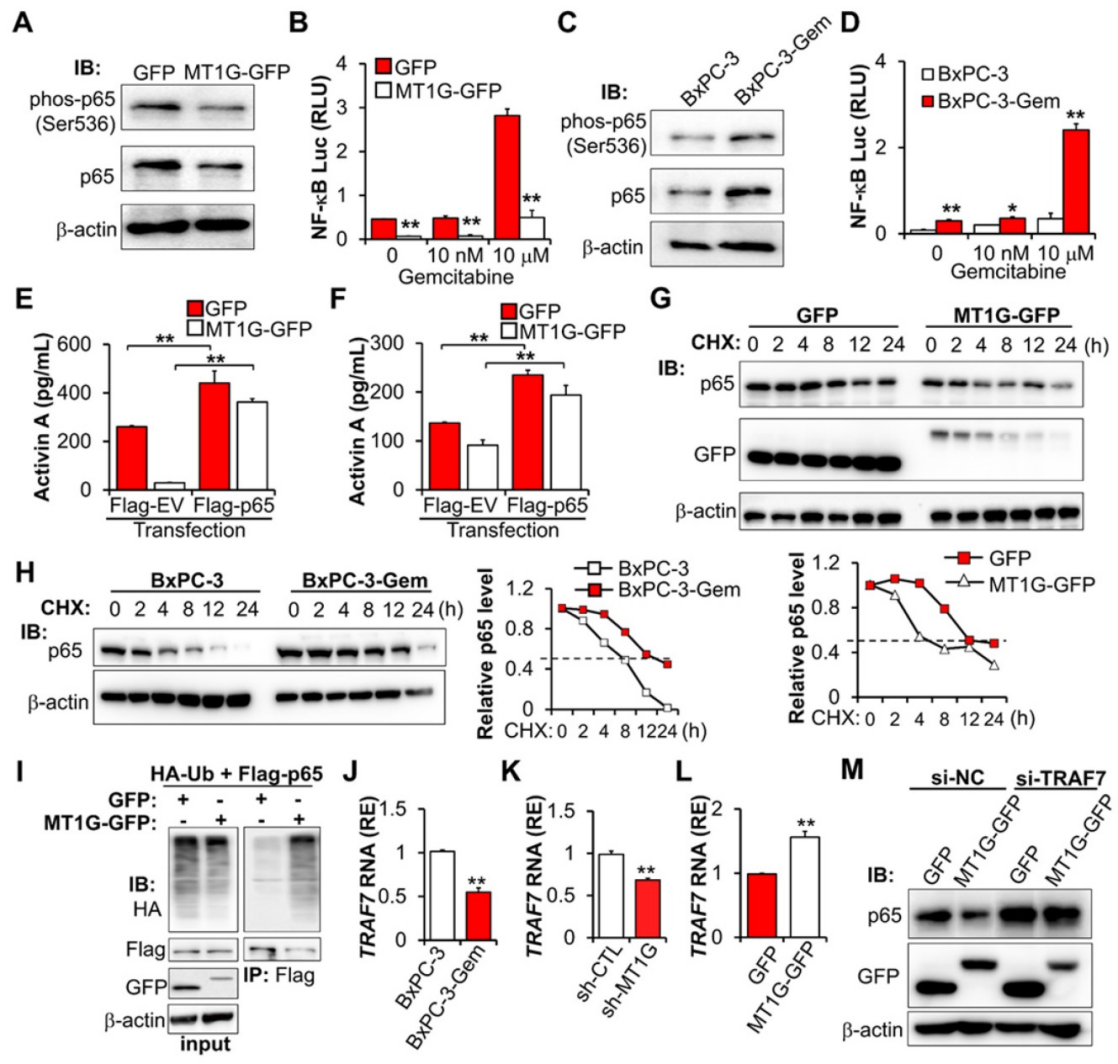
** MT1G suppresses NF-κB -activin A pathway through promoting TRAF7-mediated degradation of p65.** (**A**) IB analysis of NF-κB p65 subunit phosphorylation in MT1G overexpressing (MT1G-GFP) and control (GFP) BxPC-3-Gem cells. (**B**) Luciferase reporter assay in MT1G overexpressing and control BxPC-3-Gem cells post gemcitabine treatment for 48 hours. RLU, relative luminescence units. (**C**) IB analysis of NF-κB p65 subunit phosphorylation in BxPC-3 and BxPC-3-Gem cells. (**D**) Luciferase reporter assay to determine the activation of NF-κB in the indicated cells treated with gemcitabine for 48 hours. (**E, F**) ELISA analysis of the secreted activin A protein levels in indicated GFP or MT1G-GFP overexpressing BxPC-3-Gem (E) or PANC-1 (F) cells transfected with Flag empty vector (Flag-EV) or Flag-tagged p65 plasmid (Flag-p65) for 48 hours. (**G**, **H**) IB analysis of NF-κB p65 subunit in *MT1G* overexpressing and control BxPC-3-Gem cells (G), or BxPC-3 and BxPC-3-Gem cells (H) treated with cycloheximide (CHX) (100 μg/mL) for up to 24 hours (left). Quantification of p65 was normalized to the loading control and expressed relative to 0 hour (G bottom, H right). (**I**) PANC-1 cells were co-transfected with indicated plasmids for 48 hours and treated with MG132 (1 μM) for 12 hours and subjected to immunoprecipitation (IP) with the anti-Flag antibody, followed by IB with anti-HA and anti-Flag antibodies. Whole cell expression (input) of proteins were detected by IB with indicated antibodies. (**J-L**) RT-qPCR analysis of *TRAF7* mRNA levels in BxPC-3 and BxPC-3-Gem cells (J), *MT1G* knockdown BxPC-3 cells (K) or *MT1G* overexpressing BxPC-3-Gem cells (L). RE, relative expression. (**M**) MT1G overexpressing and control BxPC-3-Gem cells were transfected with control siRNA (si-NC) or siRNA targeting *TRAF7* (si-TRAF7) for 48 hours and subjected to IB with indicated antibodies. Data are presented as mean ± SD (n = 3) and are representative of at least three independent experiments, *P < 0.05, **P < 0.01 by two-tailed Student's *t* test.

The above results indicate that MT1G inhibits activin A secretion to suppress PDAC stemness, next we were interested to know how MT1G inhibits activin A expression. Given that MT family members are generally considered unable to regulate gene expression by directly binding to its promoter 11, we speculated that MT1G might inhibit activin A secretion by affecting NF-κB for the two reasons: 1) Activin A is a target of NF-κB 20,21. 2) NF-κB pathway was enriched by secretome analysis in *MT1G* downregulated resistant cells (Figure 3B). Thus, we analyzed the activation of NF-κB after overexpressing *MT1G* in the gemcitabine resistant PDAC cells. *MT1G* overexpression led to the diminution of the NF-κB p65 protein (Figure 5A). It also caused the decrease of phosphorylated p65 on Ser536 (Figure 5A), an indicator of NF-κB activation 22. Luciferase reporter assay confirmed that *MT1G* overexpression could decrease NF-κB activity (Figure 5B). Additionally, gemcitabine induced NF-κB activation, which could also be reduced after *MT1G* overexpression (Figure 5B). Conversely, knockdown of *MT1G* increased NF-κB p65 protein levels (both total and phosphorylated form) (Figure S5A) and enhanced basal and gemcitabine-induced activation of NF-κB (Figure S5B). Moreover, PDAC cells with low levels of endogenous *MT1G* showed increase in total and phosphorylated NF-κB p65 subunit (Figure 5C, Figure S5C) and enhanced basal and gemcitabine-induced NF-κB activity compared to the cells with high levels of *MT1G* (Figure 5D, Figure S5D). These results indicate that MT1G represses the expression of NF-κB p65 and led us to investigate whether MT1G affected activin A secretion through NF-κB inhibition. As a target of NF-κB (20, 21), overexpression of NF-κB p65 led to increased activin A secretion (Figure 5E-F). MT1G suppressed the secretion of activin A, which was blocked by p65 overexpression (Figure 5E-F), confirming that MT1G limits activin A secretion through NF-κB inhibition.

Co-IP revealed that there is no interaction between MT1G and p65 (Figure S5E), indicating that MT1G regulating NF-κB activity might through inhibiting p65 expression. However, we did not detect changes in the mRNA of p65 after overexpression or knockdown of *MT1G* (Figure S5F-G), therefore MT1G may affect p65 expression at the protein level rather than the RNA level. We determined p65 stability following treatment with protein synthesis inhibitor cycloheximide (CHX) and found that overexpression of MT1G reduced the half-life of p65 (Figure 5G). This observation was consistent with endogenous MT1G influence on p65 protein levels, showing that the half-life of p65 in gemcitabine resistant cells with low *MT1G* levels was longer than that in the sensitive cells with high MT1G levels (Figure 5H).

To further explore the mechanism of MT1G mediated p65 degradation, we first confirmed that MT1G promoted the ubiquitination of p65 (Figure 5I). Then, we re-analyzed the results of the mRNA array and found that several E3 ligases were downregulated in BxPC-3-Gem cells compared to BxPC-3 cells (Figure S5H). Among them, TRAF7 was selected (Figure S5H) as it is involved in the degradation of p65 23-25. RT-qPCR analysis confirmed the mRNA array results, showing that *TRAF7* was downregulated in BxPC-3-Gem cells (Figure 5J) and its expression was regulated by MT1G (Figure 5K-L). Furthermore, we observed a similar trend of mRNA expression of *TRAF7* and *MT1G* in PDAC clinical samples, in which both of *MT1G* and *TRAF7* were down-regulated in tumor tissues compared with the adjacent counterparts (Figure S5I, Figure 1D). To assess the role of TRAF7 in MT1G induced degradation of p65, a siRNA targeting *TRAF7* was designed to suppress the expression of *TRAF7* (Figure S5J). Immunoblotting (IB) results showed that MT1G induced degradation of p65 in BxPC-3-Gem cells was abolished after *TRAF7* knockdown (Figure 5M). Conversely, up-regulation of p65 caused by *MT1G* knockdown in BxPC-3 cells was also abolished after ectopic expression of *TRAF7* (Figure S5K). These results indicate that MT1G enhances the expression of *TRAF7* to promote ubiquitin-mediated degradation of tumor initiation gene NF-κB in pancreatic cancer cells.

### Inhibition of activin A signaling with follistatin potentiates gemcitabine efficacy

**Figure 6:**
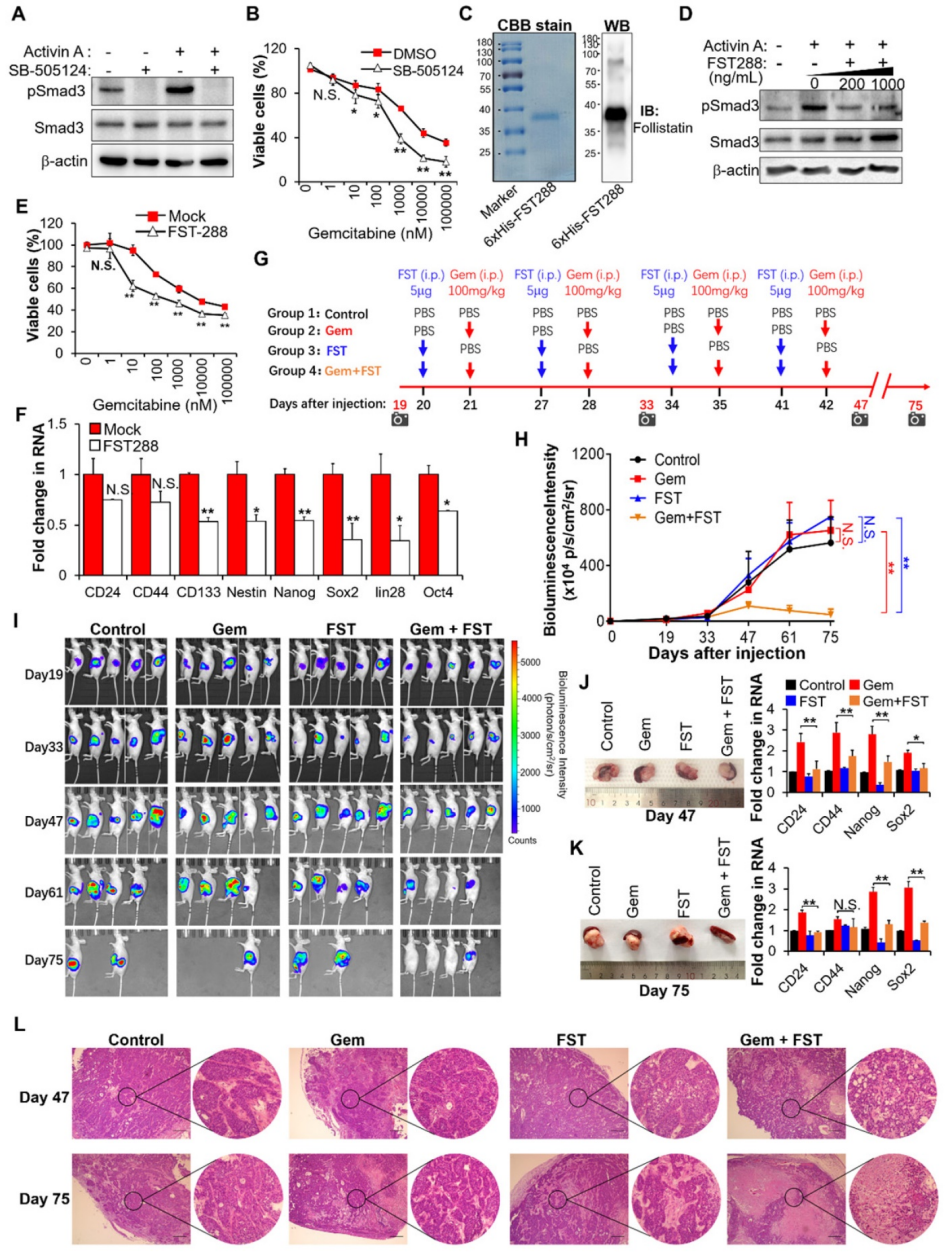
** Blockade of activin A with FST sensitizes PDAC cells to gemcitabine.** (**A**) IB analysis of Smad3 phosphorylation in BxPC-3-Gem cells treated with SB-505124 (1 μM) for 6 hours prior to treatment with activin A (10 ng/mL) for 1 hour. (**B**) MTT assay in BxPC-3-Gem cells treated with SB-505124 (1 μM) for 24 hours prior to treatment with gemcitabine for 72 hours. (**C**) Purification of recombinant His-tagged human FST 288 protein analyzed by CBB stain (left) and IB (right) analysis. (**D**) IB analysis of Smad3 phosphorylation in BxPC-3-Gem cells treated with recombinant FST (200, 1000 ng/mL) for 6 hours prior to treatment with activin A (10 ng/mL) for 1 hour. (**E**) MTT assay in BxPC-3-Gem cells treated with FST (1000 ng/mL) for 24 hours prior to treatment with gemcitabine for 72 hours. (**F**) RT-qPCR analysis of indicated CSCs markers in BxPC-3-Gem cells treated with or without FST (1000 ng/mL) for 48 hours. (**G**) Schematic description of the experiment design to determine the effect of gemcitabine in combination with FST on the growth of orthotopic BxPC-3-Gem xenografts in nude mice. IVIS imaging time points are shown (camera symbol). (**H**) Quantitative analysis of orthotopic BxPC-3-Gem tumors imaging signal intensity (photons/sec/cm^2^/steradian) over the time after injection. (**I**) Representative bioluminescent images of mice bearing orthotopic BxPC-3-Gem tumors are shown for each time point. (**J, K**) Representative images of orthotopic BxPC-3-Gem tumors with spleen (left) and RT-qPCR analysis of indicated CSC markers in tumors (right) after tumor cells injection 47 days (J) and 75 days (K). (**L**) Formalin-fixed paraffin-embedded sections of orthotopic tumors were analyzed with H&E staining. Scale bar, 200 µm. Data in (B, E, F, J, K) are presented as mean ± SD (n = 3), *P< 0.05, **P < 0.01 by two-tailed Student's *t* test. Data in (H) are presented as mean ± SD (n = 5), **P < 0.01 by one-way ANOVA, followed by Tukey test. N.S., not significant.

Based on the above results showing that activin A is the downstream target of MT1G-NF-κB regulatory axis, we tried to assess the therapeutic potential of inhibiting activin A signaling to overcome gemcitabine resistance in MT1G downregulated pancreatic cancer. We first used the small-molecule SB-505124, a potent and specific inhibitor of ALK 4/5/7 (the TGF-beta type I receptor) 26. Treatment with SB-505124 inhibited activin A-induced Smad3 phosphorylation (Figure 6A, Figure S6A) and decreased gemcitabine resistance (Figure 6B, Figure S6B). These data indicate that the inhibition of activin A signaling by ALK inhibitor enhances therapeutic efficacy of gemcitabine against pancreatic cancer. However, there are fatal challenges for clinical use of kinase-specific small-molecule inhibitors in combination with chemotherapy to prevent drug-resistant cancer stem cells, like unexpected toxicity due to on-target or off-target effects, as well as cellular pressure to compensate for the loss of function of an important kinase 27. Therefore, we considered whether follistatin (FST), an endogenous protein that binds to activin A with high affinity 28, could serve as a more tractable therapeutic approach. We first tested the efficacy of FST for gemcitabine sensitization *in vitro*. Results showed that purified recombinant human FST (30-317 aa) protein (Figure 6C) significantly inhibited activin A-induced Smad3 phosphorylation (Figure 6D, Figure S6C), decreased gemcitabine resistance (Figure 6E, Figure S6D) and reduced gene expressions of pancreatic cancer stemness markers (Figure 6F, Figure S6E) in gemcitabine resistant PDAC cells. It is apparent that FST sensitizes pancreatic cancer cells to gemcitabine.

To further evaluate the therapeutic benefit of FST in combination with gemcitabine *in vivo*, we established an orthotopic mouse model of pancreatic cancer and administered purified recombinant human FST along with gemcitabine according to the regimen outlined in Figure 6G. The *in vivo* data showed that neither FST nor gemcitabine alone influenced the growth of BxPC-3-Gem xenograft tumors compared to the control group (Figure 6H-K). However, combination gemcitabine/FST treatment enhanced the chemotherapy efficacy of gemcitabine (Figure 6H-K), with 3 out of 5 mice achieving a complete response (Figure 6I). HE staining indicated that gemcitabine treatment in combination with FST resulted in more serious cell death compared with the control, gemcitabine or FST single agent treated group, suggesting that combination of gemcitabine with FST treatment enhanced the chemotherapy efficacy of gemcitabine (Figure 6L). Of note, FST treatment alone did not decrease the expression of stemness genes in the BxPC-3-Gem xenograft as shown by RT-qPCR (Figure 6J-K) which is seemingly inconsistent with the *in vitro* data (Figure 6F). Moreover, we found that gemcitabine treatment alone significantly enhanced the stemness gene expression (Figure 6J-K), while gemcitabine and FST combination treatment counteracted it (Figure 6J-K). Similar results were seen in the PANC-1 orthotopic mice models (Figure S6F-I). These data provide strong support for the development of FST as an inhibitor of activin signaling to overcome gemcitabine resistance in MT1G down regulated pancreatic cancer.

## Discussion

**Figure 7:**
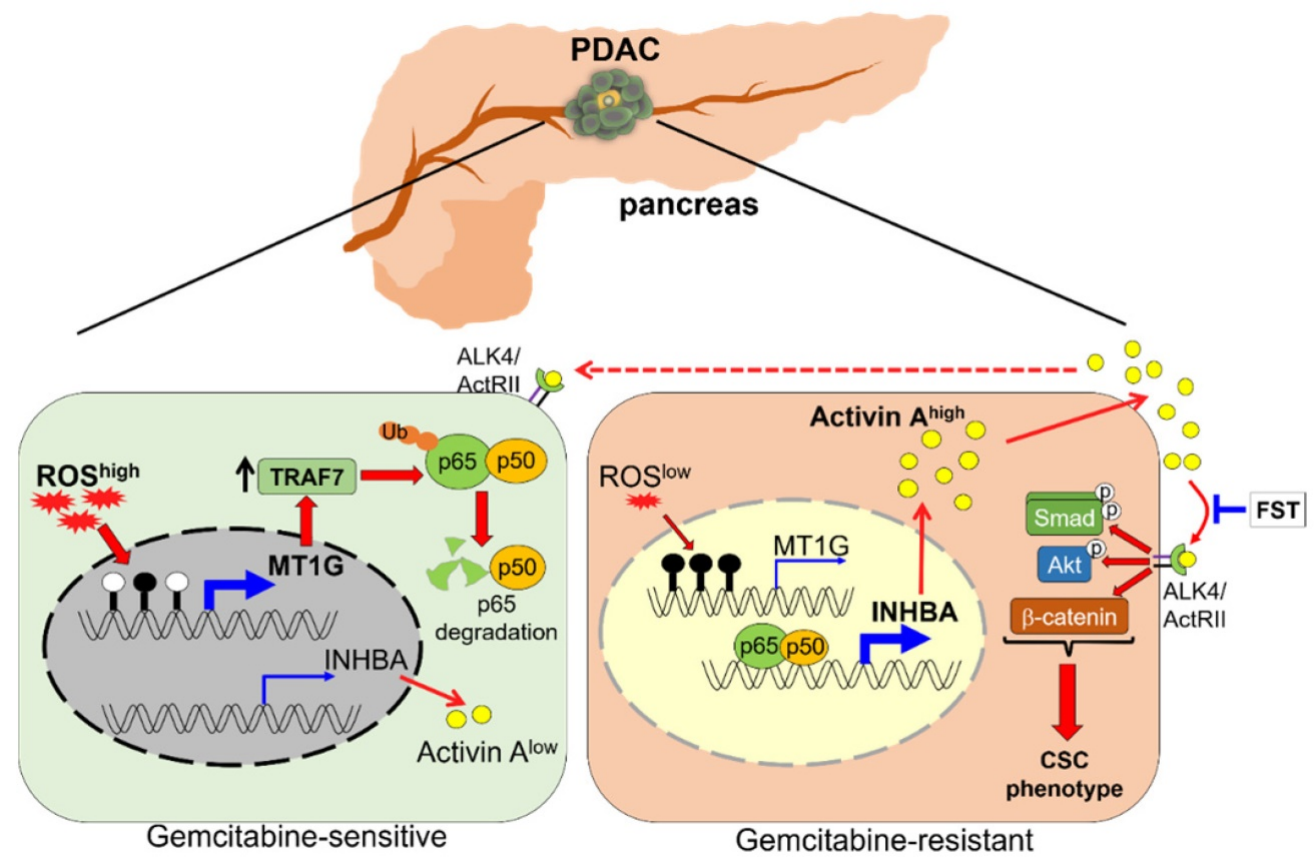
** Schematic model illustrating that downregulation of MT1G confers to chemoresistance.** In chemo sensitive PDAC cells with higher levels of ROS, the promoter of *MT1G* is hypomethylated to allow for an increased expression of *MT1G*. This leads to the upregulation of TRAF7, thereby inducing degradation of the NF-κB p65 subunit. As a result, activin A secretion is decreased. In chemoresistant PDAC cells with low levels of ROS, downregulation of MT1G due to hypermethylation results in TRAF7 decrease, which leads to NF-κB activation and activin A secretion. The secreted activin A further promotes cancer stemness in autocrine and paracrine manners, while FST can overcome chemoresistance by binding to activin A.

CSCs are highly resistant to chemotherapy and are responsible for drug resistance and cancer recurrence. Patients with PDAC are still suffering from an unfavorable prognosis, which is partially due to the standard chemotherapeutic agent gemcitabine eliminating the bulk cancer cells but leading to the enrichment of CSCs. Better understanding of the PDAC stemness regulation may provide new potential strategies for overcoming drug resistance. In this study, we identified MT1G as a critical molecule that suppressed the PDAC stemness features. MT1G repressed NF-κB activation, leading to reduced secretion of activin A. Blockade of activin A enhanced PDAC cells response to chemotherapy (Figure 7).

MT1G has been reported to suppress tumor metastasis and promote cancer cells differentiation 7,8. These two characteristics are linked with CSCs, however MT1G involvement in the regulation of cancer stemness has not been fully validated. Our results demonstrate that MT1G plays a tumor suppressor role in pancreatic cancer stem cells supported by the following facts: 1) *MT1G* expression was lower in PDAC stem cells than in bulk PDAC cells. 2) *MT1G* was hypermethylated in PDAC stem cells. Re-expression of *MT1G* triggered by ROS led to reduction of PDAC stem cells' markers. 3) Limiting diluting assay showed that overexpression of *MT1G* in drug-resistant BxPC-3-Gem or PANC-1 cells led to decrease of *in vitro* sphere formation ability and *in vivo* tumorgenicity. For the mechanism how MT1G is methylated in the drug resistance-induced cancer stem cells, we considered that long term exposure to chemo drugs such as gemcitabine might induce DNMT upregulation in drug resistant cells, as DNMT is a key player in mediating tumor suppressor genes methylation. The fact that DNA demethylating agent 5-azacytidine-mediated reprogramming can potentiate gemcitabine efficacy in drug-resistant pancreatic cancer cells 29 also support this idea. However, the exact mechanism of MT1G hypermethylation in gemcitabine resistant PDAC cells needs further investigation.

MT1G biology is incongruent amongst tumor types. MT1G is frequently downregulated in various cancers, but several upregulation cases have been reported as well 5. Our data supports that MT1G downregulation confers to chemoresistance in PDAC. Consistent with this study, multidrug resistant lung cancer cells showed downregulation of MT1G expression 30 and overexpression of MT1G sensitizes colorectal cancer cells to the chemotherapeutic agents oxaliplatin and 5-fluorouracil 31, suggesting that MT1G may contribute to chemosensitivity. However, other reports have suggested MT1G expression to be associated with drug resistance rather than drug sensitivity 32,33.

Sustained NF-κB activation is required for chemoresistance and associated with CSCs properties in pancreatic cancer 34-36. MT1G has been reported to repress NF-κB activity 31, however the underlying mechanism remains unclear. Our data show that MT1G affects the stability of NF-κB p65 subunit. It promotes p65 degradation mediated by the E3 ligase TRAF7. The fact that MT1G reduces the stability of NF-κB p65 subunit is consistent with previous observation in Mt-1 and Mt-2 double knockout (MTKO) mice, showing that MTKO mice was predominantly p50/65 heterodimer as opposed to p50/p50 in wild type mice 37. The E3 ligase TRAF7 contributes to the ubiquitination and subsequent degradation of NF-κB p65 subunit *via* K29-linkage 25. Consistently, we found that MT1G promotes p65 degradation by upregulating *TRAF7* expression. We found that MT1G upregulates *TRAF7* mRNA expression. MT is generally considered unable to regulate gene expression by directly binding to the promoter 11, however there are suggestions that MT may be working with transcription factors by serving as their source of zinc 38. Indeed, one MT family member, MT2A, has been reported binding to the promoter of NF-κB inhibitor IκB-α in association with transcription factor myeloid zinc-finger 1 (MZF1) to enhance IκB-α expression 39. Whether MT1G interacts with a transcription factor to increase *TRAF7* expression needs to be determined in the future, but our current work demonstrates that MT1G promotes the degradation of NF-κB p65 subunit by upregulating TRAF7.

Our secretome analysis revealed that activin A, the downstream target of NF-κB is the major protein secreted from the gemcitabine resistant cells. Activin A is essential for the self-renewal capacity and stemness properties of pancreatic CSCs 9. It is hardly detectable in more differentiated pancreatic cancer cells but overexpressed in pancreatic CSCs 9. This is consistent with our observation that activin A was heavily secreted from chemoresistant PDAC cells that had CSCs properties. Furthermore, we found that the secreted activin A from the gemcitabine resistant cells could educate the sensitive cells to acquire enhanced stemness properties, demonstrating that activin A can expand CSCs-like population in a paracrine manner. Therefore, targeting activin A might decrease cancer stemness properties in MT1G downregulated PDAC cells.

Our *in vivo* data demonstrate that inhibiting activin A with FST can re-sensitize resistant PDAC cells to gemcitabine in the orthotopic xenografts. This is consistent with a previous report showing that FST can increase the therapeutic index of platinum chemotherapy in lung adenocarcinoma 40. We purified FST288, instead of FST315 to block activin A signaling, because FST288 has more inhibitory effects on activin A than FST315 41. Recombinant FST as a potential agent in gemcitabine-based combination therapy has several advantages over small molecule-targeting strategies. It is superior to SB-505124 in combination with gemcitabine for the treatment of PDAC. FST exhibits strong synergy with even low concentrations of gemcitabine (nM ranges) while SB-505124 had such synergy only with higher concentrations of gemcitabine (μM ranges), suggesting that FST may reduce gemcitabine side-effects by avoiding high-dose administration of the drug. This difference may result from the difference in the mechanisms of these two molecules. FST binds to activin A and blocks its interaction with receptors, while SB-505124 is an inhibitor of activin A receptors 26. In addition, the binding affinity between FST and activin A is very strong, comparable with that of activin A and its receptors, which effectively results in a virtually irreversible reaction 42. Moreover, FST also binds to myostatin, which is a potent negative regulator of muscle mass 43. Although FST has a lower binding affinity to myostatin, it can nevertheless neutralize myostatin's functions 42. Furthermore, activin A can reproduce the biological action of myostatin on muscle tissues because it binds to the receptors shared with myostatin 44. A previous clinical study indicated that an increased circulating concentration of activin A in cancer patients may contribute to the development of cachexia 45, which is highly prevalent in PDAC patients 46. These observations further support the application of FST in gemcitabine-based therapy for PDAC.

In conclusion, our findings demonstrate that MT1G suppresses PDAC stemness by limiting activin A secretion via NF-κB inhibition. Targeting activin A with FST may provide a promising therapeutic strategy for overcoming gemcitabine resistance in PDAC.

## Materials and Methods

### Cell culture and reagents

Human PDAC cell lines BxPC-3 and previously established gemcitabine-resistant subline BxPC-3-Gem were cultured in RPMI-1640 medium (Gibco) containing 10% heat-inactivated fetal bovine serum (FBS) (Gibco) 1. Mia-PaCa2, PANC-1and HEK293T cells were cultured in Dulbecco's Modified Eagle's Medium (DMEM) (Gibco) supplemented with 10% heat-inactivated FBS. For Activin A or conditional medium (CM) treatment, cells were pretreated with medium containing 0.5% FBS for 4 hours and then replaced by medium containing 10 ng/mL activin A or indicated CM. Antibodies and other chemicals used in this study were listed in Table S3.

### Mice

All studies in animals were performed according to protocol approved by the Animal Care and Use Committee of Harbin Institute of Technology. Female athymic NU/NU nude mice (4-5 weeks old) were obtained from Beijing Vital River Laboratory Animal Technology Co., Ltd. and housed in a specific pathogen-free environment.

### *In vivo* tumorigenicity assay

The indicated number (1×10^5^, 1×10^4^, 1×10^3^) of cells were mixed with Matrigel (Becton Dickinson) and injected subcutaneously into the dorsal left or right flank of 4-5 weeks old nude mice (n = 5). After 6-12 weeks of observation, mice were sacrificed, and tumors were collected and photographed.

### Purification of human FST

Purification of human Follistatin 288 (FST, amino acid residue no. 30-317) recombinant protein was performed as previously described 47. Briefly, pET28a-FST288 plasmid was transformed into *E. coli* BL21(DE3) and induced with IPTG. The cells were then lysed in buffer (50 mM pH 8.0 Tris-HCl, 150 mM NaCl, 1% Triton-X 100, 1 mM PMSF and 1 mM EDTA) with sonication and the insoluble pellet was solubilized with buffer containing 50 mM pH 8.0 Tris- HCl, 8 M urea and 100 mM PMSF. His-tagged FST was purified with HiTrap™ Chelating HP Columns (GE Healthcare) according to the manufacturer's instructions and eluted with a buffer containing 8 M urea, then diluted (1:4) with buffer containing 200 mM pH 10.0 Tris-HCl and 2 mM DTT, finally incubated on ice for 4-5 hours. The diluted protein was dialyzed against PBS at 4 °C overnight. The purified protein was subsequently passed through Detoxi-Gel™ Endotoxin Removing Gel (Thermo Scientific) to remove bacterial endotoxins, aliquoted and stored at -80 °C until use. The purity of the recombinant FST protein (>95%) was determined by Coomassie brilliant blue (CBB) staining and further confirmed by IB with antibody against FST.

### Orthotopic pancreatic tumor model

The orthotopic pancreatic cancer mouse model was established based on previous report 48. Briefly, 4-5 weeks old nude mice were anesthetized by isoflurane inhalation. A small incision (1.5 cm) was made in the left abdominal wall. The spleen was then exteriorized along with the underlying pancreas. Approximately 5×10^5^ of BxPC3-Gem or PANC-1 cells stably expressing firefly luciferase were suspended in PBS: Matrigel (2:3) mixture and slowly injected into the tail of the pancreas. Then, the wound was closed with nylon sutures and treated with antibacterial, antimycotic cream. Growth of the pancreatic cancer xenografts was monitored by using IVIS Spectrum Imaging System (PerkinElmer) after the mice were given a single 150-mg/kg intraperitoneal (i.p.) dose of the D-luciferin (PerkinElmer). Nineteen days after tumor cell injection, the mice were divided randomly into four groups with 5 mice per group: (a) Control group with PBS injection, (b) Gem group with gemcitabine injection at 100 mg/kg body weight, (c) FST group with 5 μg/mouse recombinant FST protein injection, (d) FST + Gem group with both gemcitabine and recombinant FST protein injection. Both gemcitabine and FST protein were injected into the intraperitoneal cavity once a week for 8 weeks and FST protein was administered one day before the gemcitabine injection. On day 47 and 75 post tumor cell injection or the final monitoring by IVIS, mice were euthanized, and the pancreas of mice were harvested, photographed and frozen in -80 °C for RNA isolation.

### CM experiments

To prepare the CM for cell secretome analysis, BxPC-3 and BxPC-3-Gem cells were seeded into 10 cm dish at 5×10^6^/dish with RPMI-1640 medium containing 10% FBS. Next day, the cells were washed three times with PBS and replaced with serum-free RPMI-1640 medium. After a 24 hours culture, the supernatant was collected, centrifuged, and filtered to remove debris and detached cells, quickly frozen in liquid nitrogen and stored at -80°C until LC-MS/MS analysis. For CM treatment experiments, cells were cultured in medium containing 10% FBS for 48 hours, and the supernatant was collected, filtered, aliquoted and stored at -80°C until further use. After mixing CM with fresh medium in a 1:1 ratio, the cells were treated with this mixture for 48 hours to perform RT-qPCR, and 1 hour for IB assay. To test the role of activin A in the CM, activin A antibody (4 μg/mL) was added into this mixture and incubated at room temperature for 1 hour before CM treatment.

### Plasmid and transfection

pLKO.1 plasmid vector was used for shRNA mediated gene silencing. Sense and antisense of shRNA oligos listed in Table S4 were annealed and inserted into AgeⅠ/EcoRⅠ digested pLKO.1 vector. pLVSIN-CMV-puro plasmid vector was used for gene stable overexpression. GFP fragment was first inserted into NotⅠ/BamHⅠ digested pLVSIN-CMV-puro vector. This constructed vector (herein named pLVSIN-GFP) was also used as a control vector for overexpression experiments. Then, *MT1G* full length cDNA (without stop codon) was amplified by PCR with the primers in Table S4 and inserted into XhoⅠ/NotⅠ digested pLVSIN-GFP vector; this constructed plasmid was named pLVSIN-MT1G-GFP. pET28a+ plasmid vector was used for 6×His-tagged recombinant FST288 purification. FST (amino acid residue no. 30-317) cDNA was amplified by PCR with primers listed in Table S4 and cloned into the EcoRI/NotI site of pET28a+ vector. For transient overexpression, *TRAF7* and p65 (*RELA*) full length cDNA were amplified by PCR with primers listed in Table S4 and cloned into XhoⅠ/NotⅠ site of pCXN2-Flag vector. To generate 4×SBE Luc plasmid, a luciferase reporter containing four copies of the Smad binding site (SBE) was inserted into the KpnⅠ/XhoⅠ site of a modified pGL3-Basic vector containing thymidine kinase promoter. All these constructed plasmids were further confirmed by sequencing. For packaging lentiviruses, plasmids were transfected into cells using calcium phosphate transfection method. Transient transfection of siRNA (siRNA sequence was listed in Table S4) or plasmids were mediated by Lipofectamine 3000 (Invitrogen) reagent following the manufacturer's protocol.

### LC-MS/MS analysis

The CM prepared as described above was analyzed by Label-free LC-MS/MS. Protein in the CM were precipitated in 4 volumes of precooled acetone at -20 °C and resuspended in 8 M urea/100 mM TEAB (pH 8.0), then reduced with 10 mM dithiothreitol (DTT) at 56 °C for 30 min and alkylated with 50 mM iodoacetamide for 30 min in the dark, and finally diluted 4 times with 10 mM TEAB. Equal amount of proteins from each sample was used for tryptic digestion. After digestion, peptides were desalted using C18 columns and the desalted peptides were dried with Vacuum concentration meter. The peptide samples were dissolved in 2% acetonitrile/0.1% formic acid and analyzed using TripleTOF 5600+ mass spectrometer coupled with the Eksigent nanoLC System (SCIEX, USA). The original MS/MS file data was submitted to ProteinPilot Software v4.5 for analysis. For protein identification, the Paragon algorithm which was integrated into ProteinPilot was employed against Human proteome database for database searching. Protein abundance values were produced from LC-MS/MS datasets by using absolute protein expression (APEX) tool and scatterplot was used to assess protein abundance change between these two CMs. The latest version of the Kyoto Encyclopedia of Genes and Genomes (KEGG) database and GO categories derived from Gene Ontology (www.geneontology.org) were used for pathway analysis. Absolute log2 fold change value ≥ 2 secreted proteins were defined as differentially secreted proteins.

### RNA array and data analysis

mRNA expression microarray analysis for BxPC-3 and BxPC-3-Gem cell lines was performed in our previous study 1. After quantile normalization, data were then log transformed and displayed as scatterplot to assess gene expression variation between these two cell lines. Top 10 up and down regulated genes were listed in Table S1. For E3 ligase gene screening, differentially expressed genes (absolute log2 fold change value ≥2) were re-analyzed with human E3 ligase database (https://hpcwebapps.cit.nih.gov/ESBL/Database/E3-ligases/) to identify potential candidates for p65 degradation.

### RT-qPCR and IB analyses

Total RNAs were isolated from culture cells or frozen pancreatic tumor tissues using Trizol (Invitrogen) and reverse-transcribed (RT) into cDNA using ReverTra Ace (TOYOBO). Real-time PCR (qPCR) was performed using SYBR Premix Ex Taq (TAKARA). Detailed information about the primers is shown in Table S4. Data were normalized to the expression levels of GAPDH (Note: for PDAC clinical samples, data were normalized to 18S rRNA). Cell lysis and IB were conducted as previously described 49. Band intensity was quantified using ImageJ software (NIH).

### Stable cell lines

Knockdown and overexpression stable cell lines were established through lentiviral transduction. Briefly, lentiviral packaging mix (VSV-G plasmid and Gag-Pol plasmid) and constructed pLKO.1 or pLVSIN-CMV-puro plasmids were co-transfected into HEK293T cells. The supernatants containing lentiviruses were collected, filtered, and added into indicated cells. After 2 days of incubation with lentiviruses, the transduced cells were selected with puromycin. The knockdown efficiency or overexpression level was confirmed by RT-qPCR or IB. Cells stably expressing firefly luciferase were confirmed by measuring the luciferase activity.

### Dual luciferase reporter assay

NF-κB reporter plasmid (pNL3.2.NF-κB-RE) was obtained from Promega. 4×SBE Luc reporter plasmid containing four copies of Smad binding element (SBE) was constructed as above described. Cells were transiently co-transfected with 200 ng of reporter plasmid and 5 ng of pRL-TK renilla luciferase reporter plasmid (Toyo INK) as an internal control. For NF-κB luciferase assay, cells were treated with the indicated concentrations of gemcitabine (0, 10 nM, 10 μM). For SBE4 luciferase assay, the cells were washed with PBS twice at 24 hours post-transfection and replaced with medium containing 0.5% FBS for 4 hours, then treated with 10 ng/mL activin A. At 48 hours post-transfection, luciferase activities were measured with the Dual-Luciferase Reporter Assay system (Promega) according to the manufacturer's instructions.

### ELISA

The ELISA kit (DAC00B) (R&D system) was used to determine the concentration of activin A in the supernatant of cell cultures as per the manufacturer's instructions.

### ROS detection

The cellular ROS level was measured by incubating cells with DCFH-DA for 30 min at 37 °C. Fluorescence intensity was determined by flow cytometry. A total of 10,000 cells were analyzed per sample. For fluorescence microscopy observation, the cells were co-stained with 4'6-diamidino-2-phenylindol (DAPI).

### BSP Methylation analysis

For the analysis of* MT1G* promoter DNA methylation analysis, genomic DNA extracted from PDAC cells was modified by using EZ DNA Methylation-Gold Kit (Zymo). The bisulfite-modified DNA was used as template for nested PCR amplification by using the primers listed in Table S4. The PCR products were cloned into pEAST-T1 (TransGen Biotech) for sequencing and analyzed with DNAMAN 5 software (Lynnon Corporation).

### MACS and Flow cytometry

The CD133 MicroBead Kit (Miltenyi Biotec) was used to separate CD133^-^ and CD133^+^ cells through magnetic-activated cell sorting (MACS). To analyze the expression of CSCs surface markers in human PDAC cell lines, BxPC-3, BxPC-3-Gem, Mia-PaCa2, PANC-1 cells were harvested, washed, resuspended in 1× PBS and stained with primary (CD24, CD44, CD133) and secondary (anti-rabbit IgG, FITC conjugated or Alexa Fluor 594) antibodies that listed in Table S3. Isotype-matched irrelevant antibody (Rabbit IgG) was used as negative control. Approximately 20,000 gated events were acquired for each sample on a FACS caliber (BD Biosciences) and analyzed using FlowJo software (TreeStar, Inc.).

### MTT assay

MTT assay was performed to analyze the cell viability in response to gemcitabine. In brief, PDAC cells were seeded into 96-well plates (5,000 cells/well) and treated with various doses of gemcitabine for 72 hours, followed by the addition of MTT (5 mg/mL; Sigma). The formed MTT products were dissolved in DMSO (Sigma) and the optical density was measured at a wavelength of 490 nm using the iMark Microplate Absorbance Reader (Bio-Rad, USA). The IC_50_ value was determined through the online tool (https://www.aatbio.com/tools/ic50-calculator).

### Sphere formation and colony formation assay

For the sphere formation assay, 500 cells per well were plated onto ultra-low attachment 6-well plates (Corning) and cultured in DMEM-F12 medium (Gibco), containing 2% B27 (Gibco), 10 ng/mL of epidermal growth factor (EGF; Gibco), and 10 ng/mL of basic fibroblast growth factor (FGF; Gibco) for 14 days. Sphere formation efficiency was calculated by dividing the total number of spheres larger than 25 μm by 500 (the initial number of cells plated). For colony formation assay, BxPC-3 cells were pretreated with indicated CM in the presence or absence of neutralizing antibody of activin A (4 μg/mL) for 48 hours, then digested with trypsin, and seeded onto six-well plates (500 cells/well), followed by gemcitabine treatment. The cells were continuously cultured for 14 days without disturbance with the culture medium replaced every 5 days. The colonies were fixed in formaldehyde for 30 min, stained with 0.1% crystal violet for 30 min, and photographed.

### Histological analysis

Tumor tissues separated from mice were fixed with 4% paraformaldehyde (PFA) and subsequently embedded in paraffin and cut at 10 μm. Sections were deparaffinized and stained with hematoxylin and eosin (H&E) or prepared for immunohistochemical (IHC) stain. For H&E stain, sections were stained with Meyer Hematoxylin for 5 min at room temperature, and wash with water once, then soak in 1% HCl for 5 sec, and washed by running water for 15 min, then the section were stained with 0.5% eosin for 3 min at room temperature and washed by 100% alcohol for 1 min, then mounted for microscope observation. For IHC stain, the sections were subjected to antigen retrieval with 10 mM sodium citrate buffer (pH 6.0) in a pressure cooker. Then rinsed and blocked in 3% hydrogen peroxide to remove endogenous peroxidase. Non-specific binding sites were blocked with 2% bovine serum albumin for 30 minutes. They were incubated with CA19-9 or activin A antibody overnight at 4°C. Primary antibodies were detected with horseradish peroxidase (HRP) conjugated secondary antibody for 60 min at room temperature, followed by visualization using diaminobenzidine (DAB) substrates and mounted for microscope observation.

### Statistical analysis

Results for continuous variables are presented as means ± SD unless stated otherwise. For two-group comparison, we used two-tailed Student's *t* test. For correlation analysis, we used Pearson's correlation. For multigroup comparisons, we used ANOVA followed by Tukey test. *P* < 0.05 was considered statistically significant. All analyses were performed using SPSS v.17.0 software (SPSS Inc.).

## Supplementary Material

Supplementary figures and tables.

## Abbreviations

AKT: protein kinase B
CHX: cycloheximide
CM: conditioned medium
Co-IP: Co-immunoprecipitation
CSCs: cancer stem cells
DCFH-DA: 2',7'-Dichlorodihydrofluorescein diacetate
ELISA: enzyme-linked immunosorbent assay
FACS: fluorescence-activated cell sorting
FST: follistatin
H&E: hematoxylin and eosin
IB: Immunoblotting
IHC: Immunohistochemical
LC-MS/MS: liquid chromatography-tandem mass spectrometry
LDA: limiting dilution assay
MACS: magnetic-activated cell sorting
metallothionein: MT
NAC: N-Acetyl-L-cysteine
NF-κB: nuclear factor kappa B
PDAC: pancreatic ductal adenocarcinoma
ROS: reactive oxygen species
RT-qPCR: quantitative reverse transcription PCR
TRAF7: tumor necrosis factor receptor-associated factor 7
