# Supplementary material for: Metallothionein-1G suppresses pancreatic cancer cell stemness by limiting activin A secretion via NF-κB inhibition
Source: Theranostics. 2021 Jan 1;11(7):3196–212. doi: 10.7150/thno.51976 (PMC7847690; doi:10.7150/thno.51976)
Supplement: Supplementary file 1 — Supplementary figures and tables. [file thnov11p3196s1.pdf]

Supplemental file for

Metallothionein-1G suppresses pancreatic cancer cell stemness by limiting activin A secretion *via* NF-κB inhibition

Kai Li[1], Zhicheng Zhang[2], Yu Mei[1], Qingzhu Yang[1], Shupei Qiao[1], Cheng Ni[1], Yao Yao[1], Xinyuan Li[1], Mengmeng Li[1], Dongdong Wei[1], Wangjun Fu[1], Xuefei Guo[1], Xuemei Huang[1], Huanjie Yang[1,*]

[1]School of Life Science and Technology, Harbin Institute of Technology, Harbin, 150001, China.

[2]Department of General Surgery, Fourth Affiliated Hospital of Harbin Medical University, Harbin, 150001,   China.

*Corresponding author. E-mail: yanghj@hit.edu.cn

This supplementary material contains

1. **Figure S1.** MT1G is involved in regulation of PDAC stemness and gemcitabine resistance.
2. **Figure S2.** Expression of *MT1G* is induced by ROS.
3. **Figure S3.** Expression of *INHA* and *INHBB* in PDAC cells.
4. **Figure S4.** Effects of CM from MT1G knockdown or overexpression cells on PDAC stemness and gemcitabine resistance.
5. **Figure S5.** MT1G inhibits NF-κB activation in PDAC cells.
6. **Figure S6.** Inhibition of activin A signaling enhances the chemotherapeutic efficacy of gemcitabine in PANC-1 cells.
7. **Table S1.** Top 10 differentially expressed mRNAs in BxPC-3 and BxPC-3-Gem cells.
8. **Table S2.** Top 10 differentially secreted proteins in CM from BxPC-3 and BxPC-3-Gem cells.
9. **Table S3.** Antibodies and other chemicals used in the experiments.
10. **Table S4.** Oligos used for PCR and vector construction.

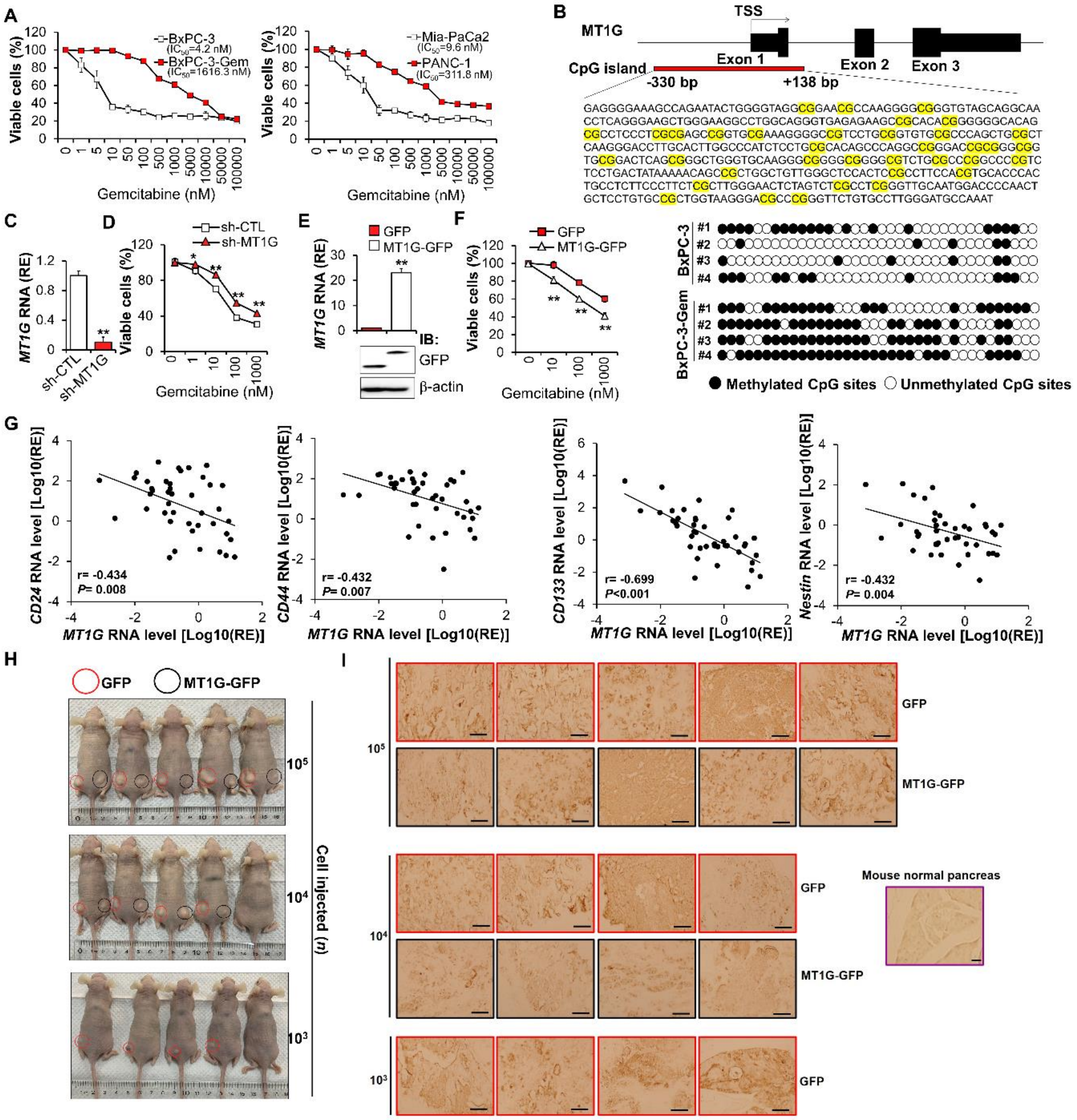

J

Limiting dilution analysis (LDA) *in vitro*

Cells		Sphere-containing wells/total wells						Sphere-initiating cell frequency	*P*-value
		Cells per well						Estimate (95% confidence interval)	
		500	100	50	10	5	1		
BxPC-3-Gem	GFP	8/8	8/8	7/8	4/12	4/20	0/20	1/24.8 (1/40.3 - 1/15.3)	0.0306
	MT1G-GFP	8/8	8/8	5/8	1/12	0/20	0/20	1/56.0 (1/94.7 - 1/33.2)	
PANC-1	GFP	8/8	8/8	8/8	12/12	20/20	5/20	1/2.41 (1/3.41 - 1/1.78)	0.006
	MT1G-GFP	8/8	8/8	8/8	12/12	13/20	2/20	1/4.95 (1/7.16 - 1/3.48)	

Figure S1. MT1G is involved in regulation of PDAC stemness and gemcitabine resistance. (**A**) Relative cell viability of indicated PDAC cell lines after treatment with gemcitabine at indicated concentrations for 72 hours. Data are presented as mean ± SD (n = 3) and gemcitabine IC_{50} concentrations for each cell lines are shown. (**B**) Schematic representation of the genomic structure of *MT1G* and DNA sequence analyzed by bisulfite genomic sequencing. The transcription start site (TSS) and position of CpG island (red bar) are presented, 36 CpG sites in the analyzed sequence are highlighted in yellow. Bisulfite sequencing PCR (BSP) analysis results of the methylation status of MT1G promoter in BxPC-3 and BxPC-3-Gem cells are shown in bottom. The numbers indicate the DNA clone numbers. Filled circles represent methylated CpGs; open circles represent unmethylated CpGs. (**C, D**) MT1G was knocked down by shRNA (sh-MT1G) in Mia-PaCa2 cells, the knockdown efficiency was determined by RT-qPCR compared with the control shRNA (sh-CTL) (C). The cells viabilities post treatment with gemcitabine for 72 hours were determined by MTT (D). (**E, F**) Overexpression of MT1G in PANC-1 cells (MT1G-GFP) was verified by RT-qPCR (E upper) and IB (E bottom) compared with control (GFP) (E). The cells viabilities post 72 hours treatment with gemcitabine were determined by MTT (F). (**G**) Pearson's correlation of mRNA expression between *MT1G* and indicated CSCs markers in PDAC and adjacent normal tissues (n = 21). (**H**) Representative images of subcutaneous xenografts with a series of diluted MT1G overexpressing (MT1G-GFP) or control (GFP) BxPC-3-Gem cells in nude mice. (**I**) CA19-9 IHC staining of each tumor separated from (H), mouse normal pancreas was used as a negative control. Scale bar, 100 µm. (**J**) *In vitro* LDA of sphere-initiating cell frequency in indicated cells. Data are presented as mean ± SD (n = 3). *$P < 0.05$, **$P < 0.01$ by two-tailed unpaired Student's *t* test.

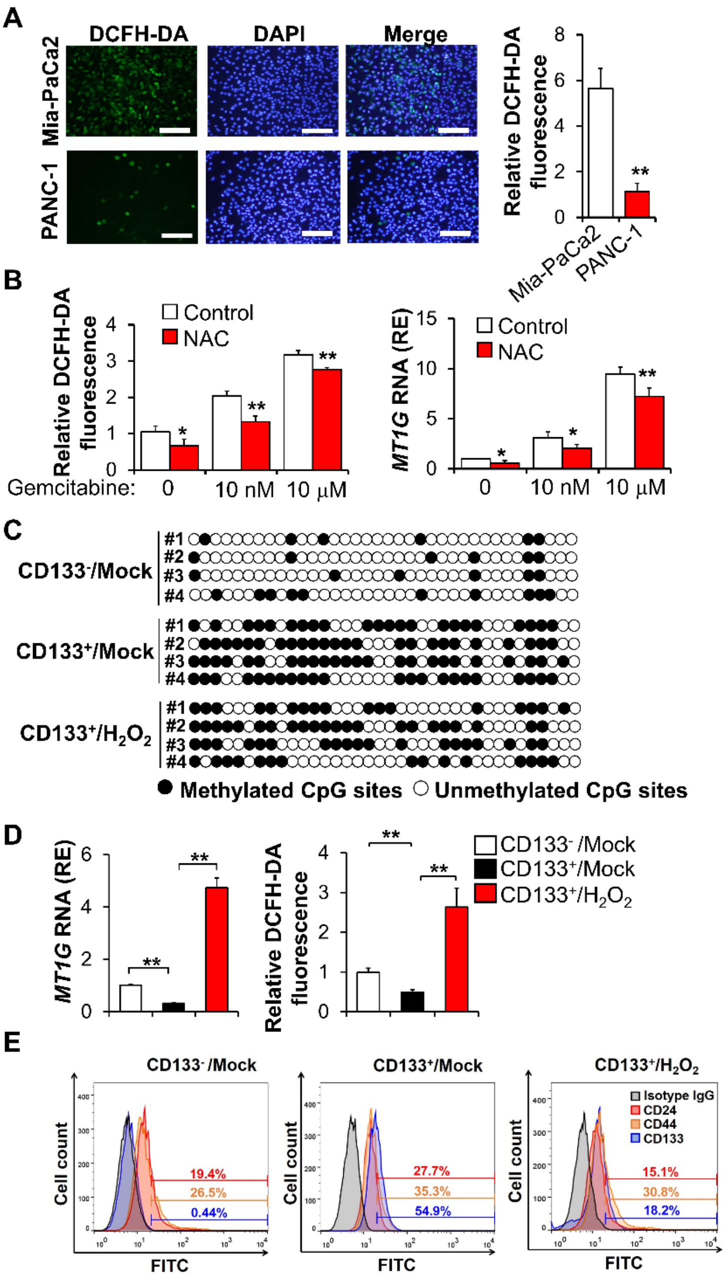

Figure S2. Expression of *MT1G* is induced by ROS. (**A**) ROS levels were evaluated using DCFH-DA staining by fluorescence microscopy (left) and flow cytometry (right) in Mia-PaCa2 and PANC-1 cells. Bar, 100 μm. (**B**) Mia-PaCa2 cells were pre-treated with NAC (5 μM) for 1 hour and then treated with indicated concentration of gemcitabine for 48 hours, relative ROS levels (up) and *MT1G* mRNA levels (down) were evaluated by flow cytometry using DCFH-DA and

RT-qPCR, respectively. RE, relative expression. (**C**) BSP analysis of the methylation status of *MT1G* promoter in separated CD133$^+$ BxPC-3 cells subpopulation were treated with or without H_2O_2 (20 μM) for 72 hours. The numbers indicate the DNA clone number. Filled circles represent methylated CpGs; open circles represent unmethylated CpGs. (**D-E**) Separated CD133$^+$ PANC-1 cells were treated with or without H_2O_2 (20 μM) for 72 hours, relative ROS levels (D left), *MT1G* expression (D right) and proteins of CSCs markers (E) were evaluated by flow cytometry using DCFH-DA (D left), RT-qPCR (D right) and Western blot (E). Data are presented as mean ± SD (n = 3). *P < 0.05, **P < 0.01 by two-tailed unpaired Student's t test.

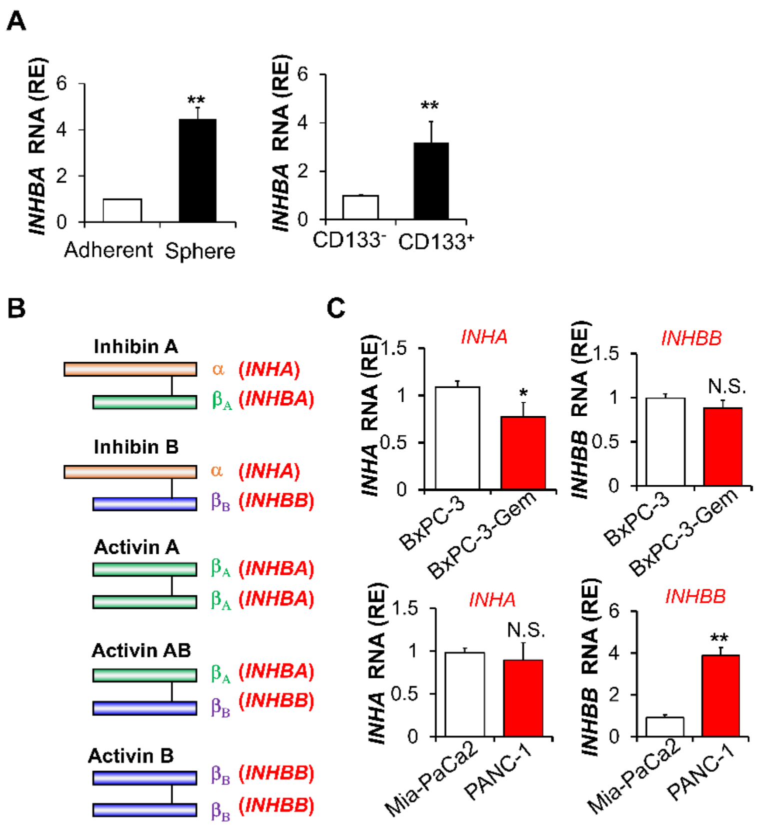

Figure S3. Expression of *INHA* and *INHBB* in PDAC cells. (A) RT-qPCR analysis of *INHBA* mRNA in BxPC-3 cells. Cancer stem cells were isolated by spheroid (left) and MACS using CD133 antibody (right). (**B**) Schematic diagram of the 1D structures of inhibin and activin. The black line between the monomers represents a disulfide bond. (**C**) RT-qPCR analysis of *INHA* and *INHBB* mRNA levels in PDAC cells. Data are presented as mean ± SD (n = 3). *$P < 0.05$, **$P < 0.01$ *vs* BxPC-3 or Mia-PaCa2 group by two-tailed unpaired Student's *t* test. N.S., not significant.

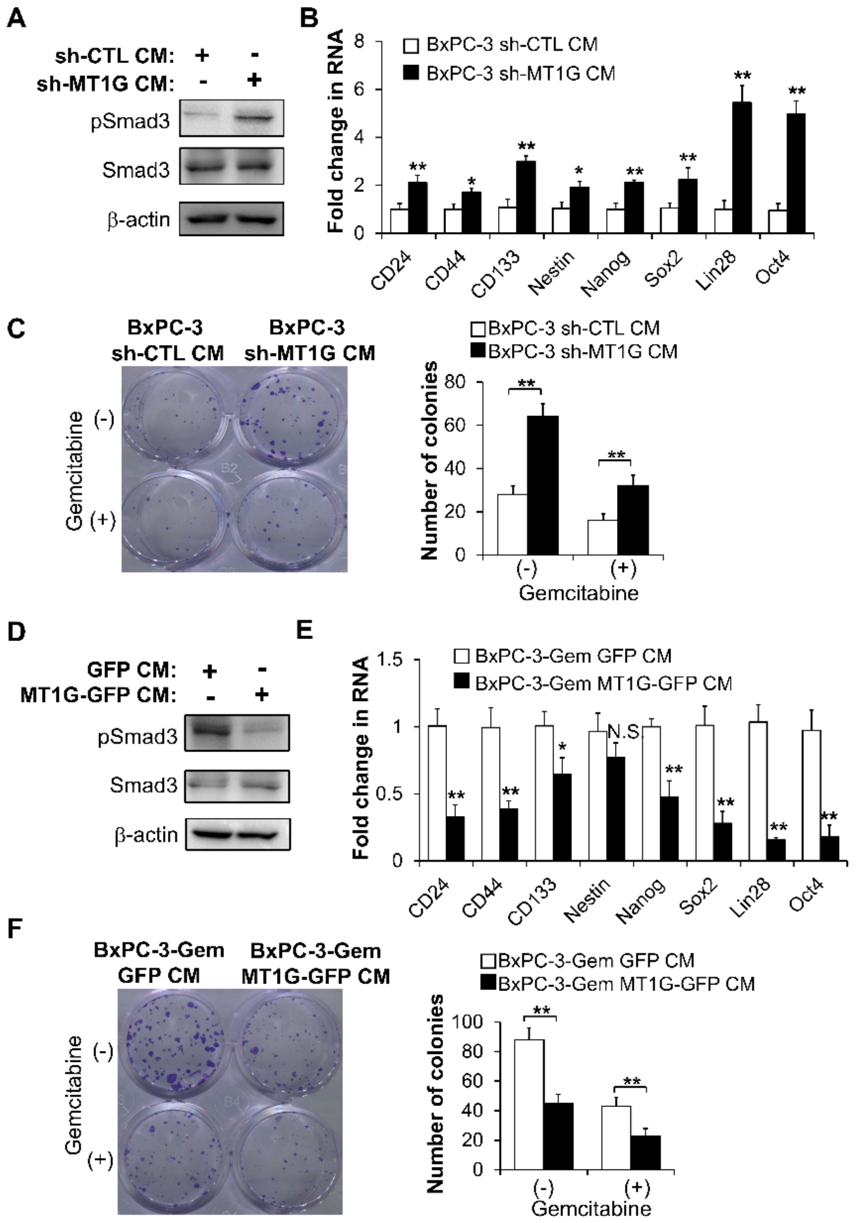

Figure S4. Effects of CM from MT1G knockdown or overexpression cells on PDAC stemness and gemcitabine resistance. BxPC-3 cells were treated with CM from control and MT1G knockdown BxPC-3 (A-C), or from control and MT1G overexpressing BxPC-3-Gem cells (D-F) for 1 hour (A, D) or 48 hours (B-C, E-F). Smad activation was measured by IB with the indicated antibodies (A, D), mRNA expressions of indicated CSC markers were analyzed by RT-qPCR (B, E), cancer cell survival in response to gemcitabine (1 nM) was determined by colony formation

assay (C, F). Data are presented as mean ± SD (n = 3). *$P < 0.05$, **$P < 0.01$ by two-tailed unpaired Student's *t* test. N.S., not significant.

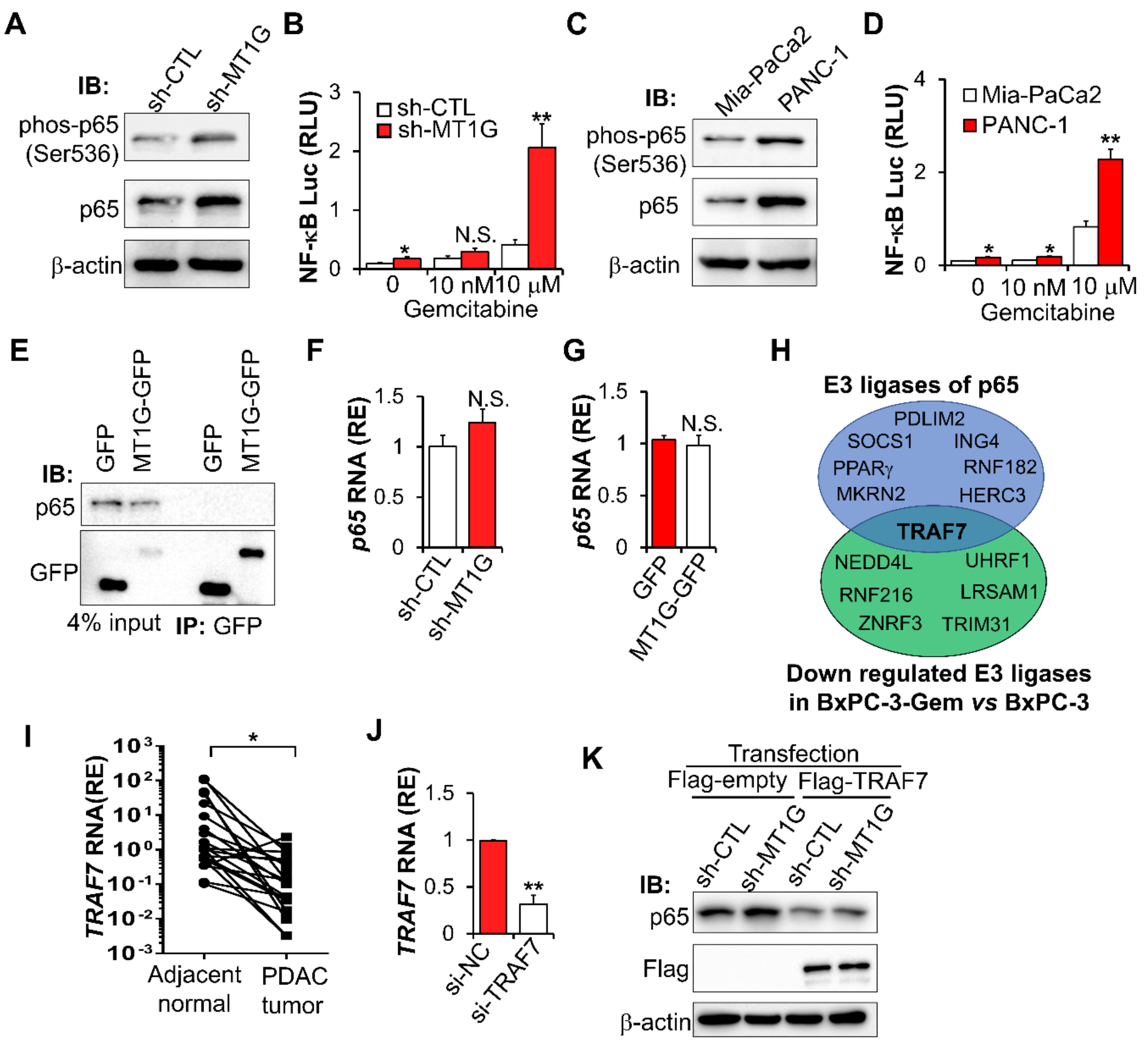

Figure S5. MT1G inhibits NF-κB activation in PDAC cells. (**A**) IB analysis of NF-κB p65 subunit phosphorylation in *MT1G* knockdown (sh-MT1G) or control (sh-CTL) BxPC-3 cells. (**B**) Activation of NF-κB in sh-MT1G or sh-CTL BxPC-3 cells treated with gemcitabine for 48 hours was measured by luciferase reporter assays. RLU, relative luminescence units. (**C**) IB analysis of NF-κB p65 subunit phosphorylation in Mia-PaCa2 and PANC-1 cells. (**D**) Activation of NF-κB

in the indicated cells treated with gemcitabine for 48 hours was measured by luciferase reporter assays. **(E)** Cell lysates from control (GFP) or *MT1G* overexpressing (MT1G-GFP) BxPC-3-Gem cells were immunoprecipitated (IP) with the anti-GFP antibody, followed by IB with anti-p65 and anti-GFP antibodies. Whole cell expression (input) of proteins were detected by IB with indicated antibodies. (**F**, **G**) RT-qPCR analysis of p65 mRNA levels in *MT1G* knockdown BxPC-3 cells (F) or *MT1G* overexpressing BxPC-3-Gem cells (G). **(H)** Venn diagram represents the intersection of reported E3 ligases of p65 and downregulated E3 ligases in BxPC-3-Gem compared with BxPC-3 cells. (**I**) RT-qPCR analysis of *TRAF7* in PDAC and adjacent normal tissues (n = 21). (**J**) Knockdown efficiency of siRNA for *TRAF7* was analyzed by RT-qPCR. RE, relative expression. NC, negative control. (**K**) *MT1G* knockdown (sh-MT1G) or control (sh-CTL) BxPC-3 cells were transfected with Flag empty vector or Flag-tagged TRAF7 overexpression vector for 48 hours and subjected to IB with indicated antibodies. Data are presented as mean ± SD (n = 3). *$P < 0.05$, **$P < 0.01$ by two-tailed unpaired Student's t test. N.S., not significant.

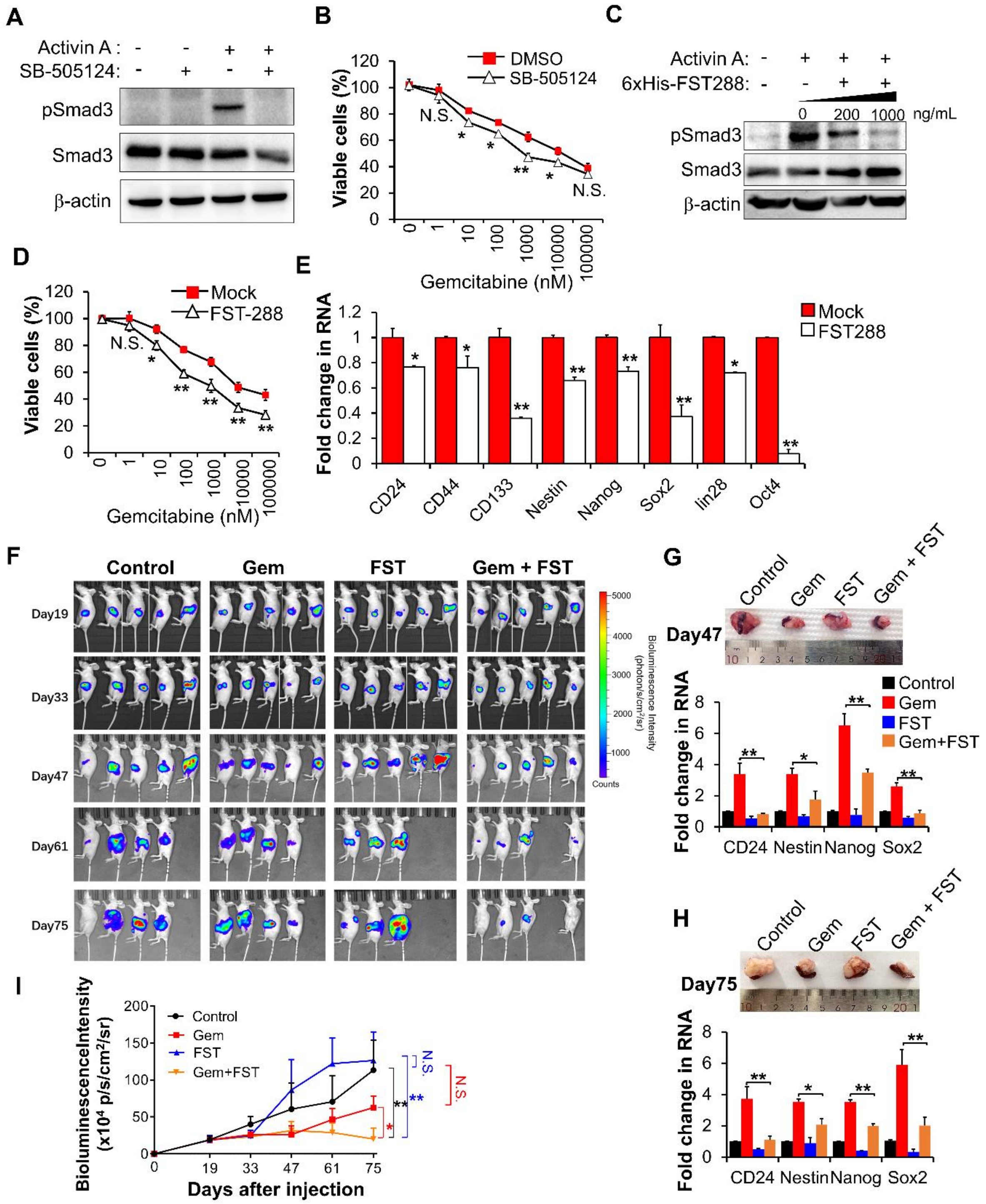

Figure S6. Inhibition of activin A signaling enhances the chemotherapeutic efficacy of gemcitabine in PANC-1 cells. (**A**) IB analysis of Smad3 phosphorylation in PANC-1 cells treated with SB-505124 (1 μM) for 6 hours prior to treatment with Activin A (10 ng/mL) for 1 hour. (**B**) MTT assay in PANC-1 cells treated with SB-505124 (1 μM) for 24 hours prior to treatment with

gemcitabine for 72 hours. (**C**) IB analysis of Smad3 phosphorylation in PANC-1 cells treated with indicated concentrations of FST for 24 hours prior to treatment with Activin A (10 ng/mL) for 1 hour. (**D**) MTT assay in PANC-1 cells treated with FST (1000 ng/mL) for 24 hours prior to treatment with gemcitabine for 72 hours. (**E**) RT-qPCR analysis of indicated CSC markers in PANC-1 cells treated with or without FST (1000 ng/mL) for 48 hours. (**F**) Representative bioluminescent images of mice bearing orthotopic PANC-1 tumors are shown for each time point. (**G, H**) Representative images of orthotopic PANC-1 tumors with spleen (upper) and RT-qPCR analysis of indicated CSC markers in tumors (bottom) at Day 47 (H) and Day 75 (I) after tumor cells injection. (**I**) Quantitative analysis of orthotopic PANC-1 tumors imaging signal intensity (photons/sec/cm^2/steradian) over the time after injection. Data in (B, D, E, G, H) are presented as mean ± SD (n = 3), *$P < 0.05$, **$P < 0.01$ by two-tailed unpaired Student's *t* test. Data in (I) are presented as mean ± SD (n = 5), *$P < 0.05$, **$P < 0.01$ by one-way ANOVA, followed by Tukey test. N.S., not significant.

Table S1. Top 10 differentially expressed mRNAs in BxPC-3 and BxPC-3-Gem cells.

Gene Symbol	Seqname	mRNA Expression Raw Intensities		Log2 Fold change
		BxPC-3-Gem	BxPC-3	(BxPC-3-Gem/BxPC-3)
CACNA1C	NM_001129839	187.7147	5	5.250
SSX3	NM_021014	1059.818	30.83232	5.102
SSX4B	NM_001040612	602.6346	22.26644	4.745
SSX2	NM_175698	3463.712	141.5403	4.620
SSX2B	NM_001164417	4229.917	176.551	4.599
CYP17A1	ENST00000369887	141.2338	7.103916	4.254
MMP3	NM_002422	1728.485	106.871	4.046
LAMA2	NM_000426	82.17658	5	4.037
SSX2	NM_003147	13738.11	856.1769	4.029
POTEE	NM_001083538	167.5361	10.36382	3.962
LUM	NM_002345	5	82.89243	-4.051
NLRP7	ENST00000340844	10.43099	203.5297	-4.286
CLDN3	NM_001306	26.49765	533.8471	-4.332
FFAR2	NM_005306	28.48151	899.0275	-4.980
MSTN	NM_005259	6.550027	233.7105	-5.157
MT1G	NM_005950	377.2028	22624.14	-5.906
CCBL2	NM_001008661	1377.006	102262.1	-6.215
PIK3C2B	NM_002646	78.69128	7244.306	-6.525
ADAMTS4	NM_005099	28.21544	2618.911	-6.536
HOXA3	NM_030661	19.07395	3389.23	-7.473

Table S2. Top 10 differentially secreted proteins in CM from BxPC-3 and BxPC-3-Gem cells.

Protein ID	Coverage	Mass	#Unique Peptide	BxPC-3 Abund	BxPC-3-Gem Abund	Log2 Fold change (BxPC-3-Gem /BxPC-3)
sp|P08476|INHBA	0.4507	47441.9	16	276910	24694179	6.479
sp|Q92896|GSLG1	0.1289	134550.6	9	461380	27014131	5.872
sp|P50281|MMP14	0.1598	65893.4	6	3992373	1.06E+08	4.727
sp|Q14114|LRP8	0.0737	105632.4	4	166423	3939355	4.565
sp|P20908|CO5A1	0.0985	183559.3	8	419441	8716128	4.377
sp|P15514|AREG	0.119	27895.1	4	275272	5634323	4.355
sp|P19367|HXK1	0.1461	102485.1	8	3172732	52691854	4.054
sp|O43795|MYO1B	0.0951	131983.9	7	3783276	61542345	4.024
sp|O00267|SPT5H	0.0589	120998.9	4	2204775	30623370	3.796
sp|P03956|MMP1	0.7505	54006.6	62	1.32E+08	1.71E+09	3.691
sp|Q7Z406|MYH14	0.0496	227868.6	6	9150369	758929.5	-3.592

sp|Q9Y2W2|WBP11	0.0952	69997	3	14077477	1098034	-3.680
sp|Q9BT78|CSN4	0.1552	46268.4	4	34797216	2291064	-3.925
sp|O95433|AHSA1	0.1982	38274.1	4	13897305	911345.6	-3.931
sp|Q15785|TOM34	0.2654	34559	5	6803643	430207.3	-3.983
sp|P78318|IGBP1	0.1917	39221.6	5	25783726	1449292	-4.153
sp|Q8WWM7|ATX2L	0.0521	113372.9	3	15303565	829969.1	-4.205
sp|Q9H2G2|SLK	0.0972	142694	7	6802197	368211.9	-4.207
sp|Q16630|CPSF6	0.1198	59209.3	4	17146791	844304.8	-4.344
sp|Q9NQW7|XPP1	0.0995	69917.2	4	8544713	335010.5	-4.673

Table S3. Antibodies and other chemicals used in the experiments.

REAGENT or RESOURCE	SOURCE	IDENTIFIER
**Antibodies**		
anti-Smad2	Cell Signaling Technology	Cat. # 12747T
anti-Smad3	Cell Signaling Technology	Cat. # 12747T
anti-Phospho-Smad2 (Ser465/467)	Cell Signaling Technology	Cat. # 12747T
anti-Phospho-Smad3 (Ser423/425)	Cell Signaling Technology	Cat. # 12747T
anti-Smad4	Cell Signaling Technology	Cat. # 12747T
anti-NF-κB p65	Cell Signaling Technology	Cat. # 8284
anti-phospho-NF-κB p65 (ser536)	Cell Signaling Technology	Cat. # 3033
anti-Akt	Cell Signaling Technology	Cat. # 4685S
anti-phospho-Akt (ser473)	Cell Signaling Technology	Cat. # 4060S
anti-CD24	Abcam	Cat. # ab202073
anti-CD44	Abcam	Cat. # ab189524
anti-CD133	Abcam	Cat. # ab216323
anti-Rabbit IgG, FITC Conjugated	Thermo Fisher	Cat. # F-2765
anti-Rabbit IgG, Alexa Fluor 594	Thermo Fisher	Cat. # A-21207
anti-Activin A	Thermo Fisher	Cat. # PA5-47004
anti-beta-catenin	Proteintech	Cat. # 51067-2-AP
anti-Follistatin	Santa Cruz	Cat. # sc-365003
anti-CA19-9	Zsbio Commerce Store	Cat. # ZM-0021
anti-β-actin	Sigma	Cat. # A1978
**Chemicals**		
MG-132	MedChemExpress	Cat. # 133407-82-6
Proteinase Inhibitor Cocktail	MedChemExpress	Cat. # HY-K0010
cycloheximide (CHX)	MedChemExpress	Cat. # HY-12320
SB-505124	MedChemExpress	Cat. # HY-13521
B-27^{TM} Supplement	Thermo Fisher	Cat. # 17504044
EGF	Thermo Fisher	Cat. # PHG0315
FGF-Basic	Thermo Fisher	Cat. # AA1-155
DCFH-DA (2',7'-Dichlorofluorescin diacetate)	Beyotime	Cat. # S0033

NAC (N-Acetyl-L-cysteine)	Beyotime	Cat. # S0077
BAY 11-7082	Beyotime	Cat. # S1523-10mg
Puromycin	Santa Cruz	Cat. # sc-108071
Polybrene	Santa Cruz	Cat. # sc-134220
DMSO (dimethyl sulfoxide)	Sigma	Cat. # D2650
DAPI (4',6-Diamidino-2-phenylindole dihydrochloride)	Sigma	Cat. # 9542
Matrigel	Becton Dickinson	Cat. # 356234
Gemcitabine	LC Laboratories	Cat. # G-4177
D-luciferin	PerkinElmer	Cat. # 122799
Human recombinant Activin A	R&D system	Cat. # 338-AC-010

Table S4. Oligos used for PCR and vector construction.

Oligonucleotide	Sequence (5'-3')
**siRNA**	
si-NC (negative control)	Sense: UUUUCCGAACGUGUCACGUTT<br>Antisense: ACGUGACACGUUCGGAGAATT
si-TRAF7	Sense: GGGCACACGUUCUGUAGGA<br>Antisense: UCCUACAGAACGUGUGCCC
**shRNAs construction oligos**	
pLKO.1-CTL-shRNA	Sense: CCGGTTCTCCGAACGTGTCACGTCTCGAGACGTGACACGTTCGGAGAATTTTTG<br>Antisense: AATTCAAAAATTCTCCGAACGTGTCACGTCTCGAGACGTGACACGTTCGGAGAA
pLKO.1-MT1G-shRNA	Sense: CCGGGCTCCCAAGTACAAATAGACTCGAGTCTATTTGTACTTGGGAGCTTTTTG<br>Antisense: AATTCAAAAAGCTCCCAAGTACAAATAGACTCGAGTCTATTTGTACTTGGGAGC
**Plasmids construction primers**	
pLVSIN-MT1G-GFP	Forward: AAACTCGAGATGGACCCCAACTGCTCCTGTGC<br>Reverse: AAAGCGGCCGCAGGCGCAGCAGCTGCACTTCT
Flag-TRAF7	Forward: AAACTCGAGATGAGCTCAGGCAAGAGTGCCCGC<br>Reverse: AAAGCGGCCGCTTAGCAAGTCCAAACCTTCACAGT
Flag-p65	Forward: AAACTCGAGATGGACGAACTGTTCCCCCTC

	Reverse: AAAGCGGCCGCTTAGGAGCTGATCTGACTCAG
4xSBE-Luc	Sense: CGTCTAGACTGCCGTCTAGACTTAGTACGTCTAGACTGCCGTCTAGACTTAGTACC
	Antisense: TCGAGGTACTAAGTCTAGACGGCAGTCTAGACGTACTAAGTCTAGACGGCAGTCTAGACGGTAC
pET28a-FST288	Forward: AAAGAATTCGGGAACTGCTGGCTCCGTCAA
	Reverse: AAAGCGGCCGCTCAGTTGCAAGATCCGGAGTGCT
**RT-qPCR primers**	
Sox2	Forward: GGGAAATGGGAGGGGTGCAAAAGAGG
	Reverse: TTGCGTGAG TGTGGATGGGATTGGTG
Oct4	Forward: CGCCGTATGAGTTCTGTG
	Reverse: GGTGATCCTCTTCTGCTTC
Nanog	Forward: AAGAACTCTCCAACATCCTGAAC
	Reverse: CCTTCTGCGTCACACCATT
Lin28	Forward: AAAGGAGACAGGTGCTAC
	Reverse: ATATGGCTGATGCTCTGG
CD24	Forward: CTCCTACCCACGCAGATTTATTC
	Reverse: AGAGTGAGACCACGAAGAGAC
CD44	Forward: CTGCCGCTTTGCAGGTGTA
	Reverse: CATTGTGGGCAAGGTGCTATT
CD133	Forward: TTGTGGCAAATCACCAGGTA
	Reverse: TCAGATCTGTGAACGCCTTG
Nestin	Forward: CCTCAAGATGTCCCTCAGCC
	Reverse: GGAGCAAAGATCCAAGACGC
MT1G	Forward: CTTCTCGCTTGGGAACTCTA
	Reverse: AGGGGTCAAGATTGTAGCAAA
p65	Forward: CTGCCGGGATGGCTTCTAT
	Reverse: CCGCTTCTTCACACACTGGAT
TRAF7	Forward: TCTGCGCTCCACATTCTCAC
	Reverse: ACGGGACACTTCTCTGACTT
INHBA	Forward: GGAGAACGGGTATGTGGAGA
	Reverse: AATCTCGAAGTGCAGCGTCT
INHA	Forward: CAAGTATGAGACAGTGCCC
	Reverse: GCCATCTATTTCCCAACTCTG
INHBB	Forward: TAGGTTGAGTCGCCGCTCGC
	Reverse: GTCAAAGTGTACTTCCAGGA
GAPDH	Forward: TGCACCACCAACTGCTTAGC
	Reverse: GGCATGGACTGTGGTCATGAG
18S rRNA	Forward: AACTTTCGATGGTAGTCGCCG
	Reverse: CCTTGGATGTGGTAGCCGTTT
**BSP analysis primers**	
MT1G promoter	Forward 1: GATTAAAGGATTGAGGTGGGA

Reverse 1: CCTCAAACCCAAAAACACTCT
Forward 2: GAGGGGAAAGTTAGAATATTGG
Reverse 2: ATTTAACATCCCAAAACACAAA
